# Supplementary material for: The real-time infection hospitalisation and fatality risk across the COVID-19 pandemic in England
Source: Nat Commun. 2024 May 31;15:4633. doi: 10.1038/s41467-024-47199-3 (PMC11143367; doi:10.1038/s41467-024-47199-3)
Supplement: Supplementary file 1 — Supplementary Information [file 41467_2024_47199_MOESM1_ESM.pdf]

**Supplementary Figure 1**

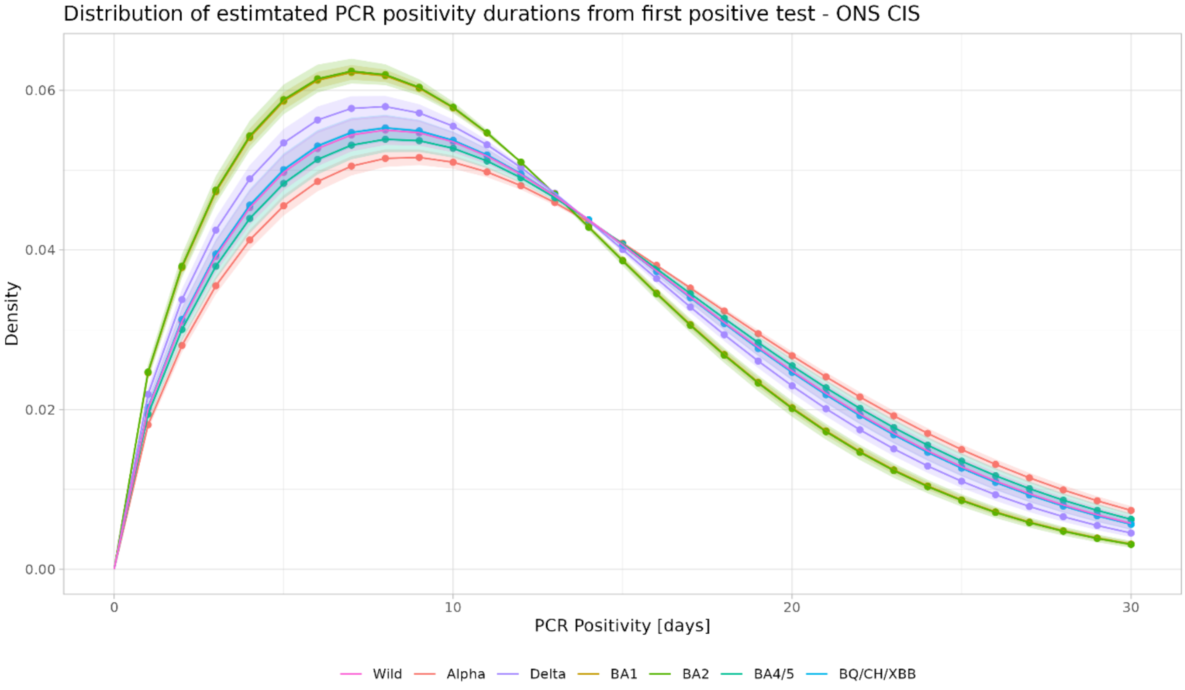


*Supplementary Figure 1 A density plot of the estimated PCR positivity duration for Wild type, Alpha, Delta, Omicron BA.1, Omicron BA.2, Omicron BA.5, and Omicron BQ/CH/XBB variants.*

## Supplementary Table 1

| PCR Positivity | | | | | | | |
|---|---|---|---|---|---|---|---|
| Variant | N | Mean | Standard Deviation | 25th Percentile | Median | 75th Percentile | 90th Percentile |
| Wild type | 1,995 | 12.589 | 8.722 | 7.419 | 12.615 | 19.169 | 26.039 |
| Alpha | 7,822 | 13.426 | 9.294 | 7.907 | 13.443 | 20.428 | 27.748 |
| Delta | 7,951 | 11.964 | 8.273 | 7.038 | 11.967 | 18.183 | 24.700 |
| Omicron BA.1 | 30,219 | 11.150 | 7.709 | 6.558 | 11.150 | 16.943 | 23.015 |
| Omicron BA.2 | 13,554 | 11.123 | 7.689 | 6.541 | 11.121 | 16.899 | 22.955 |
| Omicron BA.4/BA.5 | 12,543 | 12.882 | 8.903 | 7.574 | 12.878 | 19.569 | 26.581 |
| Omicron BQ/CH/XBB | 15,074 | 11.393 | 8.679 | 7.383 | 12.554 | 19.076 | 25.912 |

*Supplementary Table 1 The mean, standard deviation, and percentiles for Wild type, Alpha, Delta, Omicron BA.1, Omicron BA.2, Omicron BA.4/5 and Omicron BQ/CH/XBB variants.*

**Supplementary Figure 2**

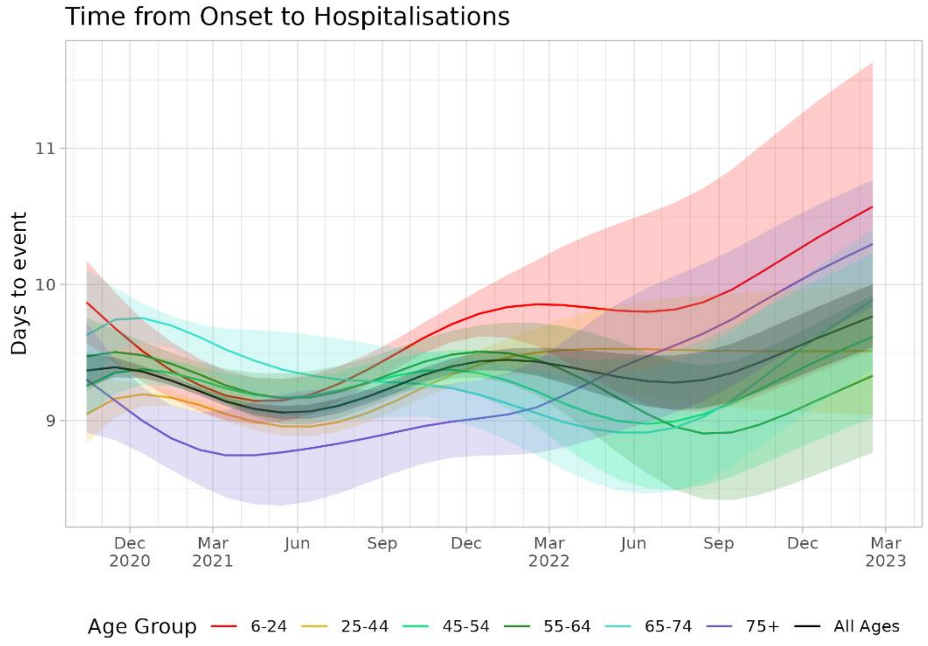


*Supplementary Figure 2 The smoothed modelled mean time from symptom onset to hospitalisation with 95% CrIs by age group using a doubly interval censored model adjusting for right truncation.*

## Supplementary Figure 3

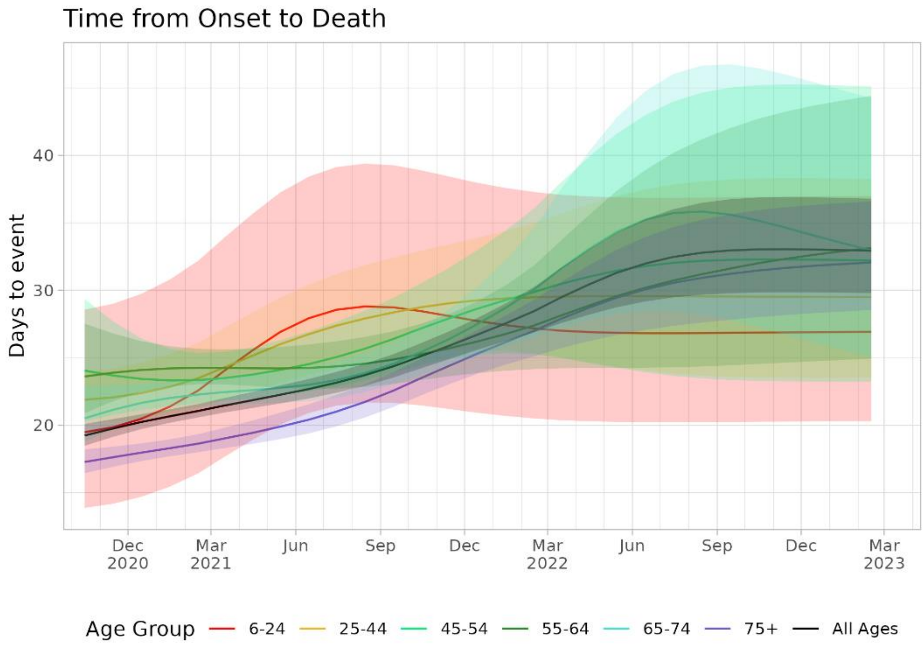


*Supplementary Figure 3 The smoothed modelled mean time from symptom onset to death with 95% CrIs by age group using a doubly interval censored model adjusting for right truncation.*

**Supplementary Figure 4**

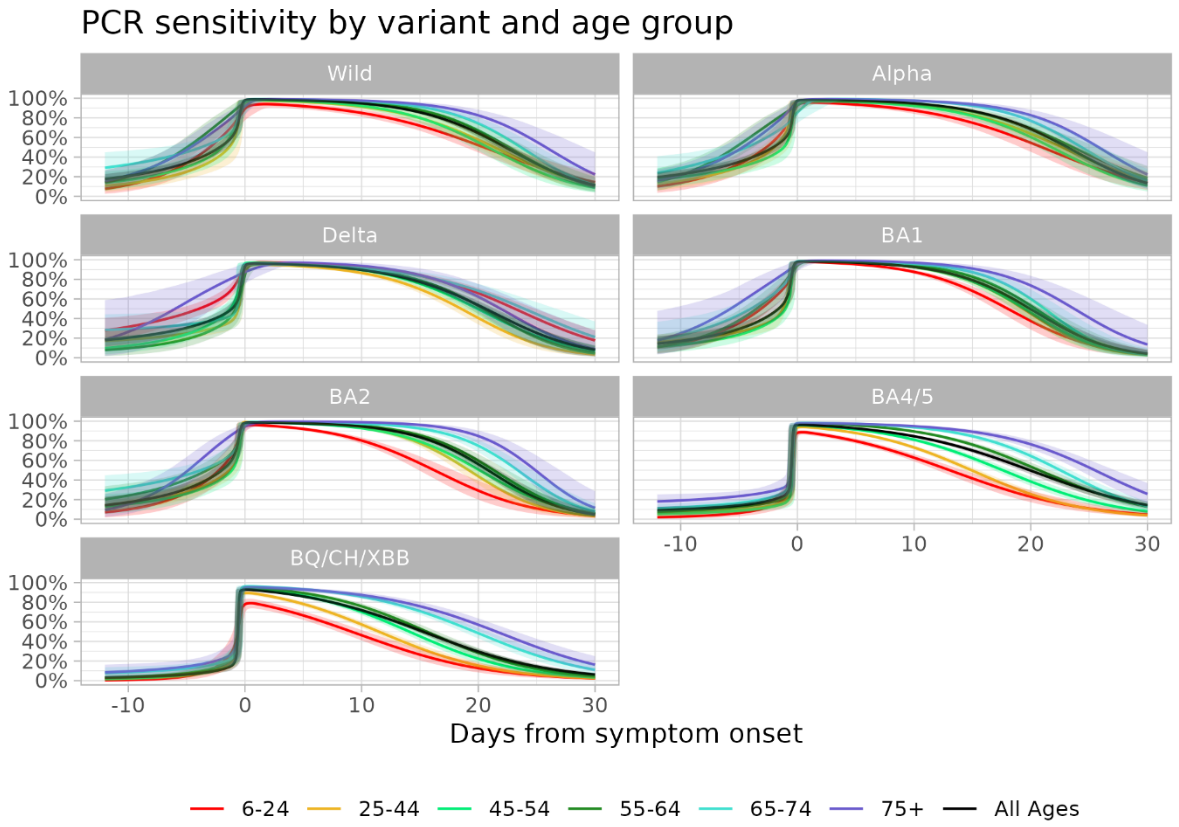


*Supplementary Figure 1 PCR Sensitivity from symptom onset date by age group (with 95% credible intervals) facetted by variant. Negative values indicate pre-symptomatic.*

## Supplementary Figure 5

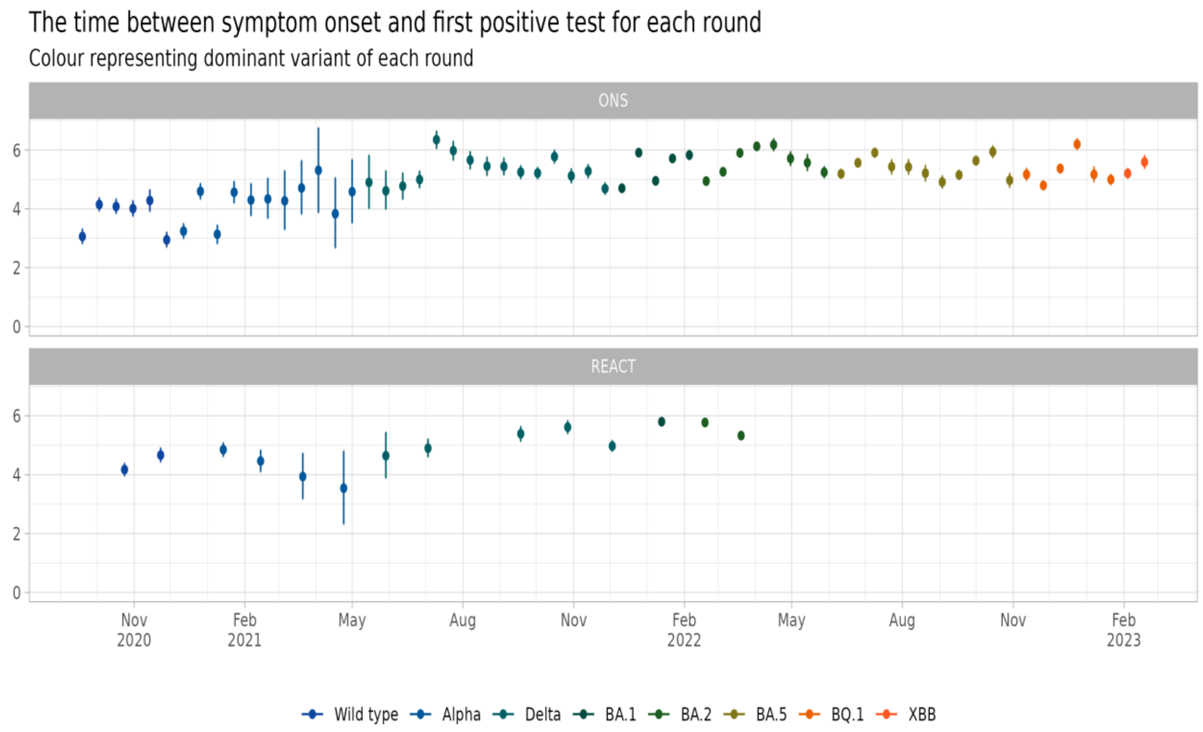


*Supplementary Figure 5 The posterior median time between symptom onset and first positive test for the Office of National Statistics Coronavirus Infection Survey and REACT 1 survey (REACT rounds were informed by data from the Office of National Statistics Coronavirus Infection Survey), with 95% credible intervals.*

## Supplementary Figure 6

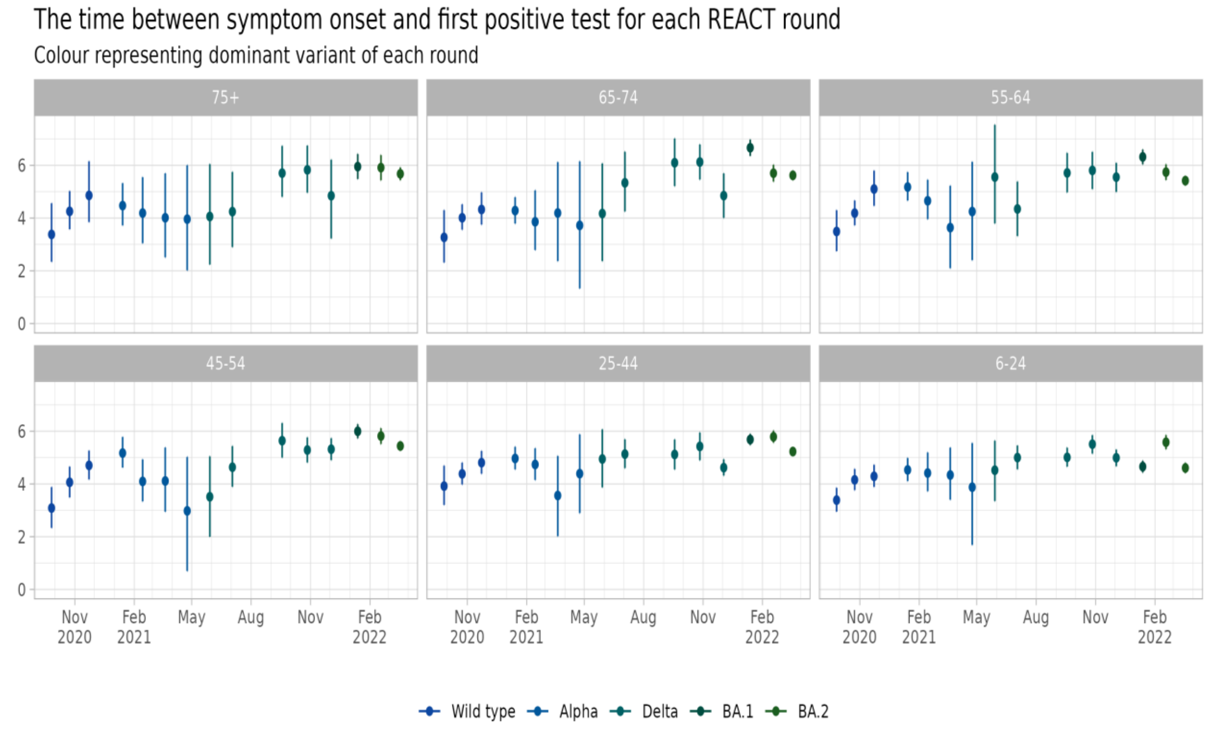


*Supplementary Figure 6 The posterior median time between symptom onset and first positive test by age group for the REACT 1 survey (REACT rounds were informed by data from the Office of National Statistics Coronavirus Infection Survey), with 95% credible intervals.*

## Supplementary Figure 7

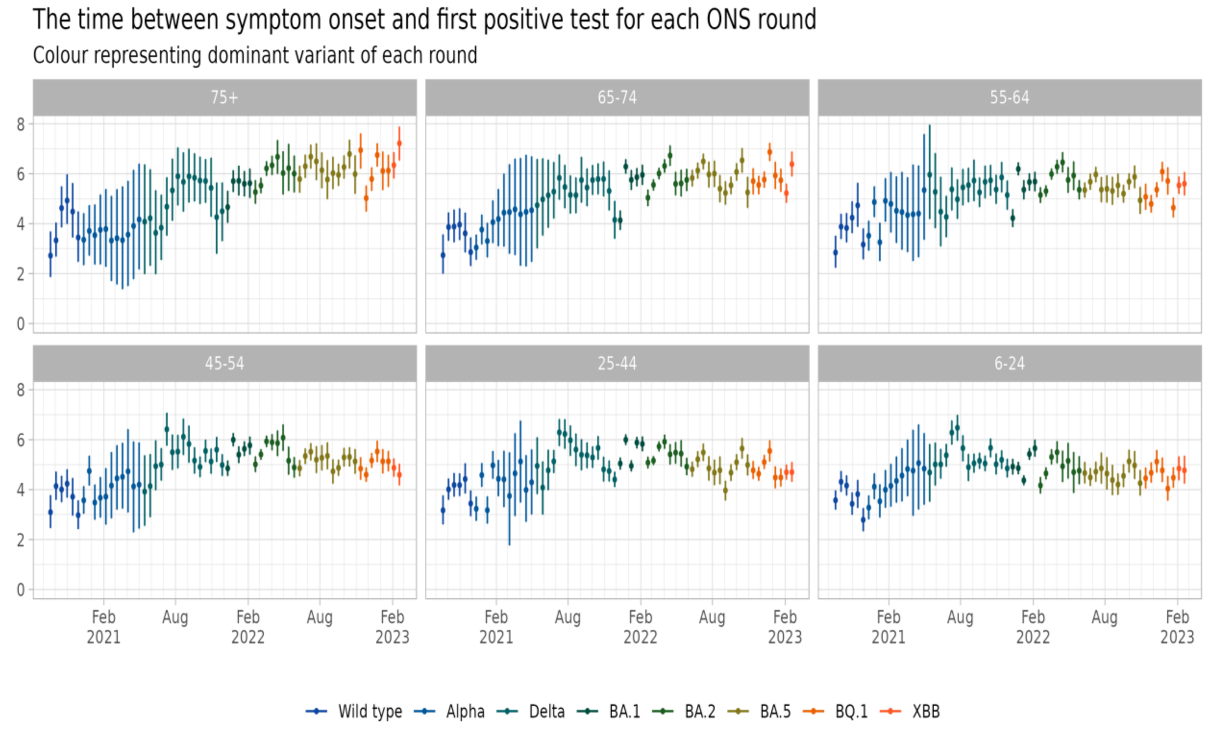


*Supplementary Figure 7 The posterior median time between symptom onset and first positive test by age group for the Office of National Statistics Coronavirus Infection Survey, with 95% credible intervals.*

**Supplementary Figure 8**

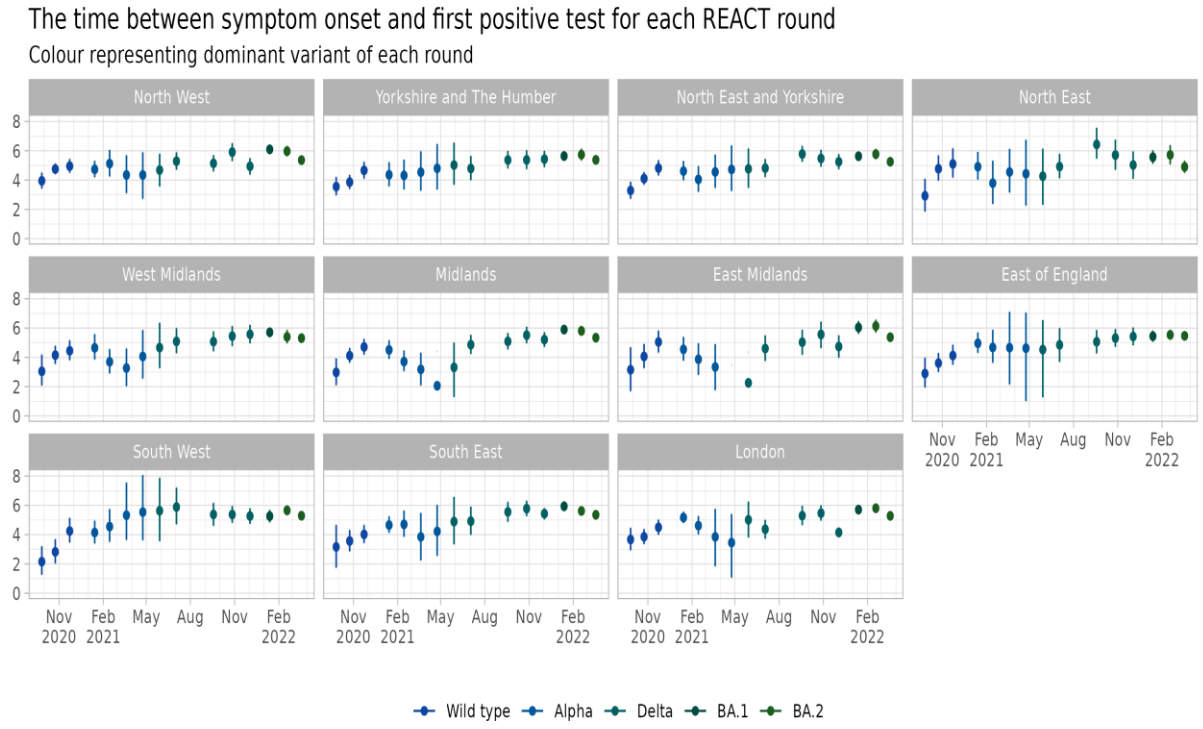


*Supplementary Figure 8 The posterior median time between symptom onset and first positive test by region for the REACT 1 survey (REACT rounds were informed by data from the Office of National Statistics Coronavirus Infection Survey), with 95% credible intervals.*

## Supplementary Figure 9

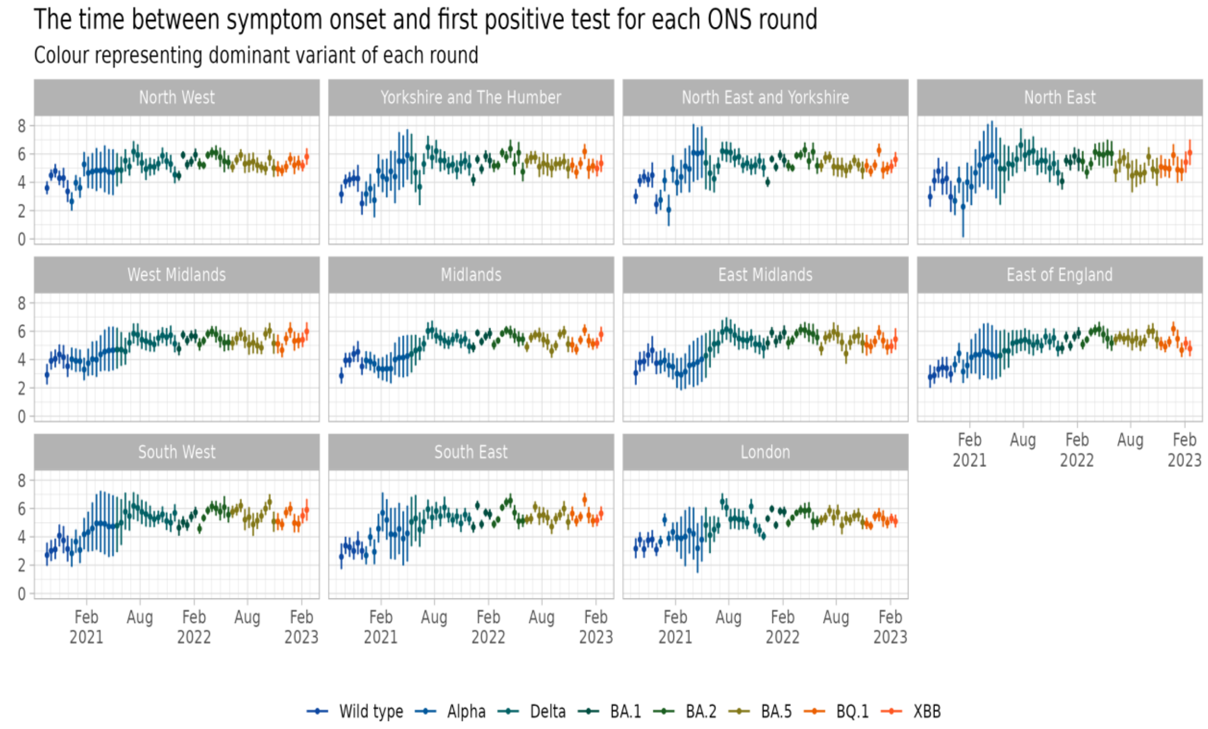


*Supplementary Figure 9 The posterior median time between symptom onset and first positive test by region for the Office of National Statistics Coronavirus Infection Survey, with 95% credible intervals.*

**Supplementary Figure 10**

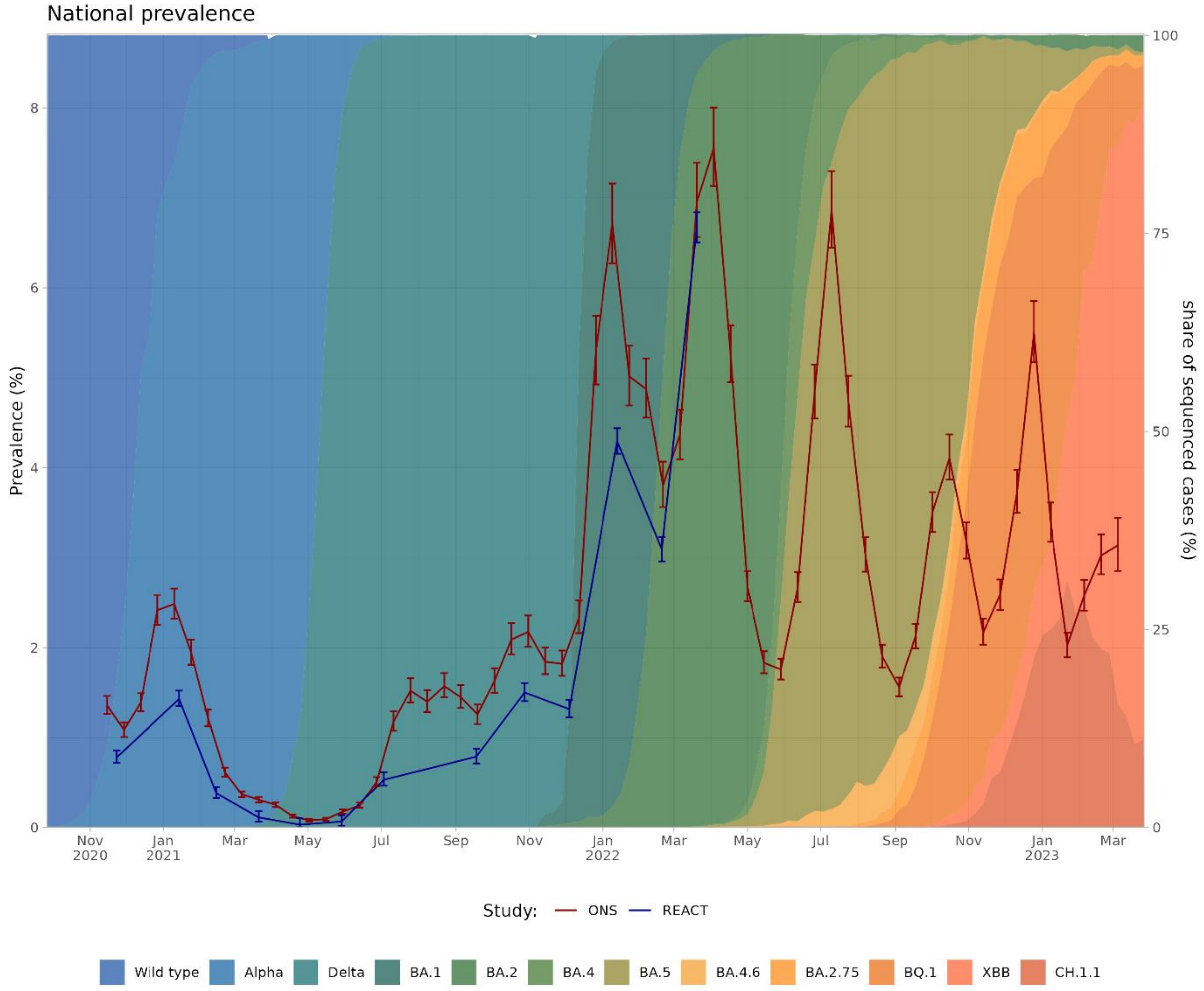


*Supplementary Figure 10 Prevalence estimates derived from the Office of National Statistics Coronavirus Infection Survey and the REACT 1 antigen survey run by Imperial College London in conjunction with Ipsos MORI for England from November 2020 until March 2023 (the REACT study concluded in March 2022).*

**Supplementary Figure 11**

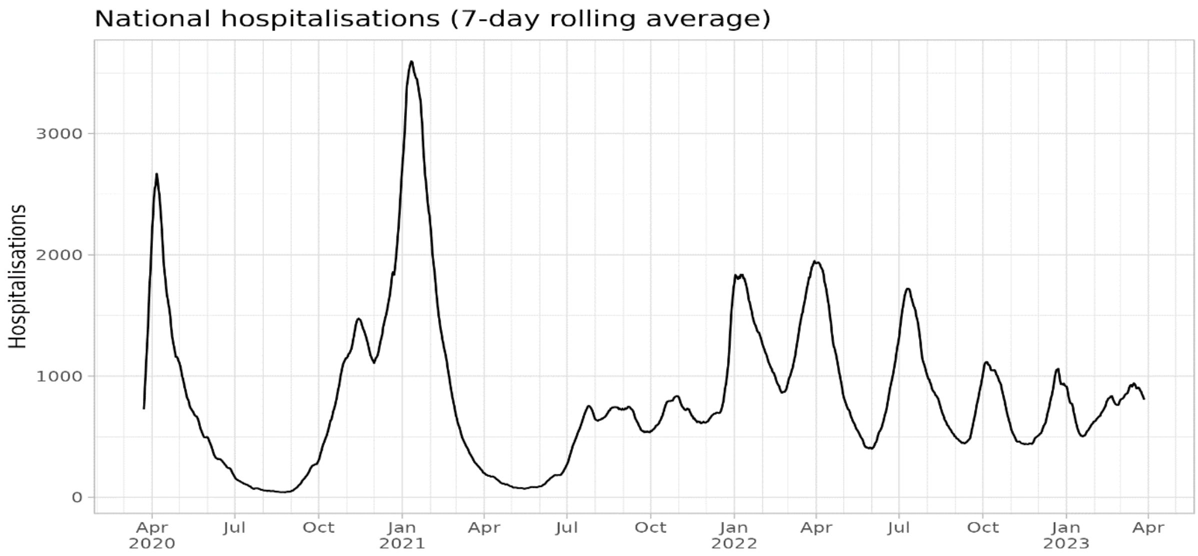


*Supplementary Figure 11 COVID-19 hospital admissions confirmed through RT-PCR testing in England from March 2020 until April 2023, sourced from NHSE&I situational reports.*

## Supplementary Figure 12

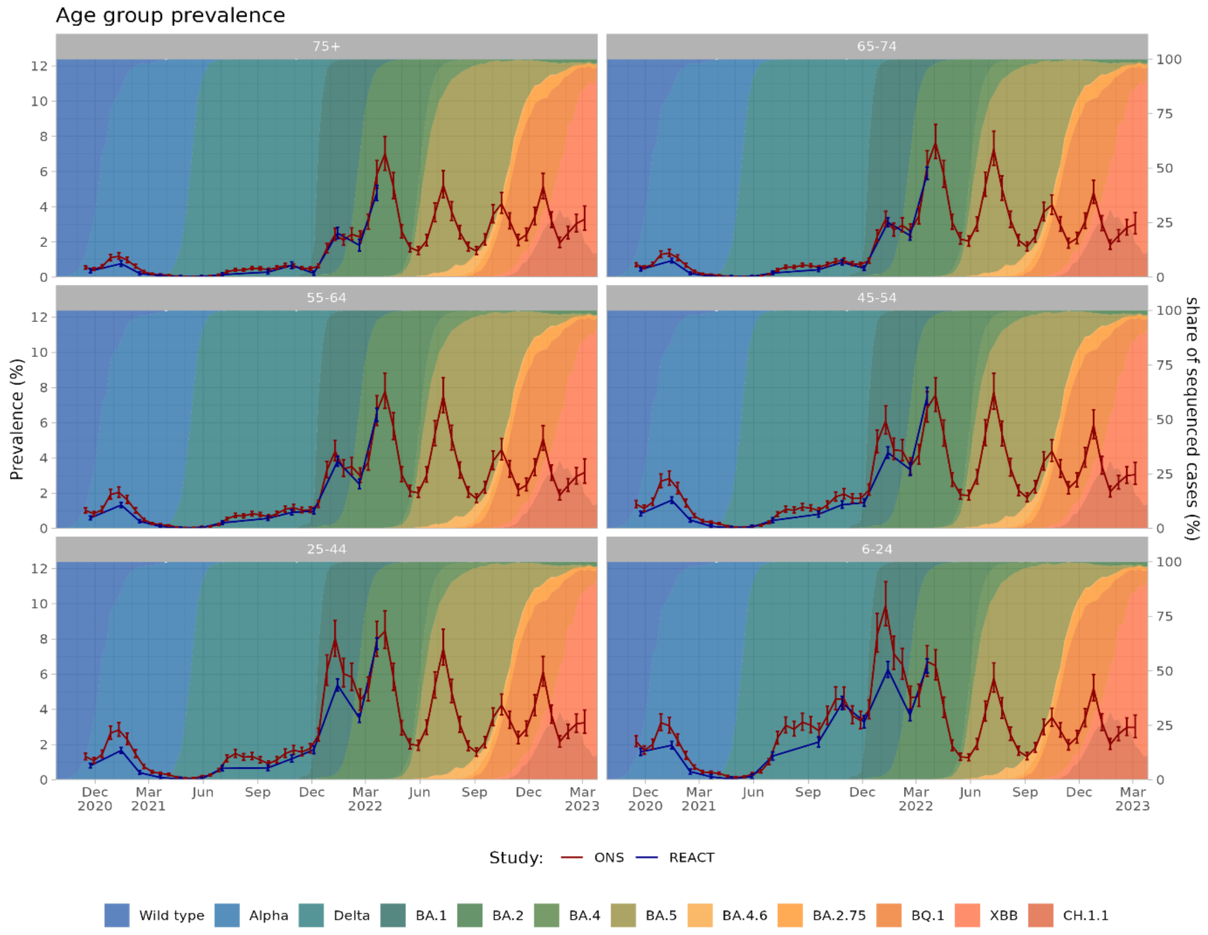


*Supplementary Figure 12 Prevalence estimates derived from the Office of National Statistics Coronavirus Infection Survey and the REACT 1 antigen survey run by Imperial College London in conjunction with Ipsos MORI for age groups in England from November 2020 until March 2023 (the REACT study concluded in March 2022).*

## Supplementary Figure 13

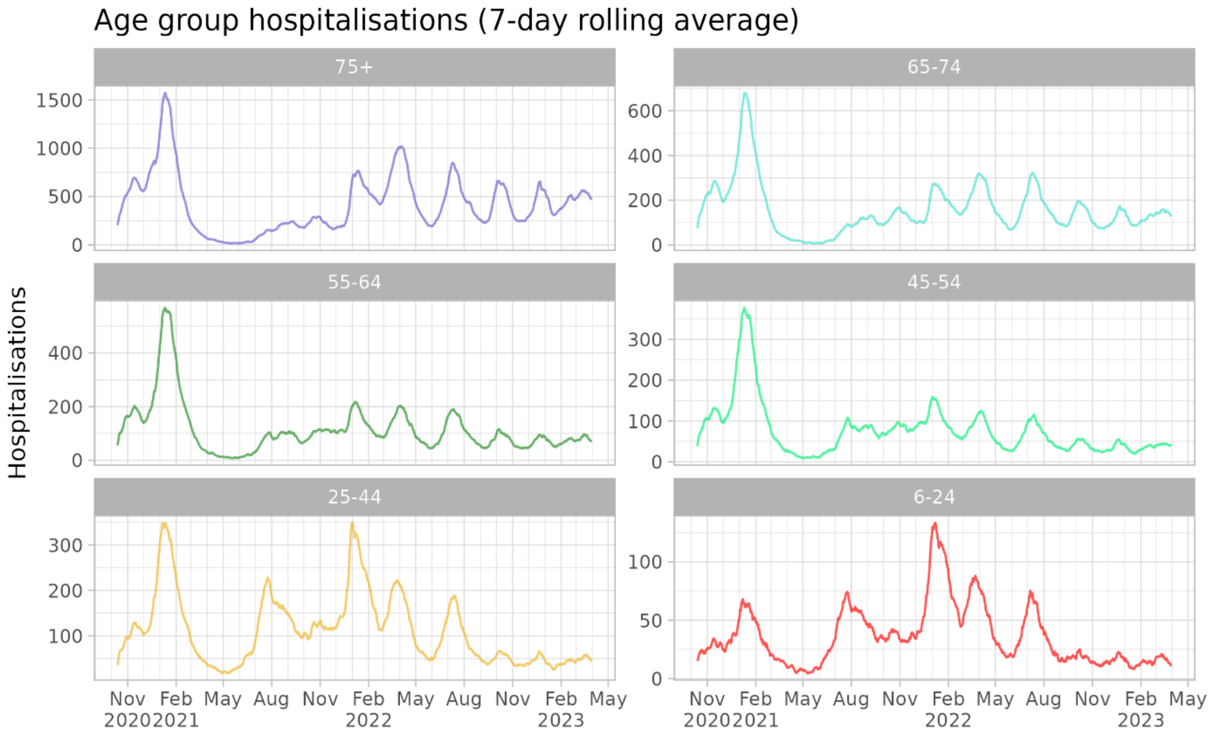


*Supplementary Figure 13 COVID-19 hospital admissions confirmed through RT-PCR testing by age group in England from March 2020 until April 2023, sourced from NHSE&I situational reports.*

## Supplementary Figure 14

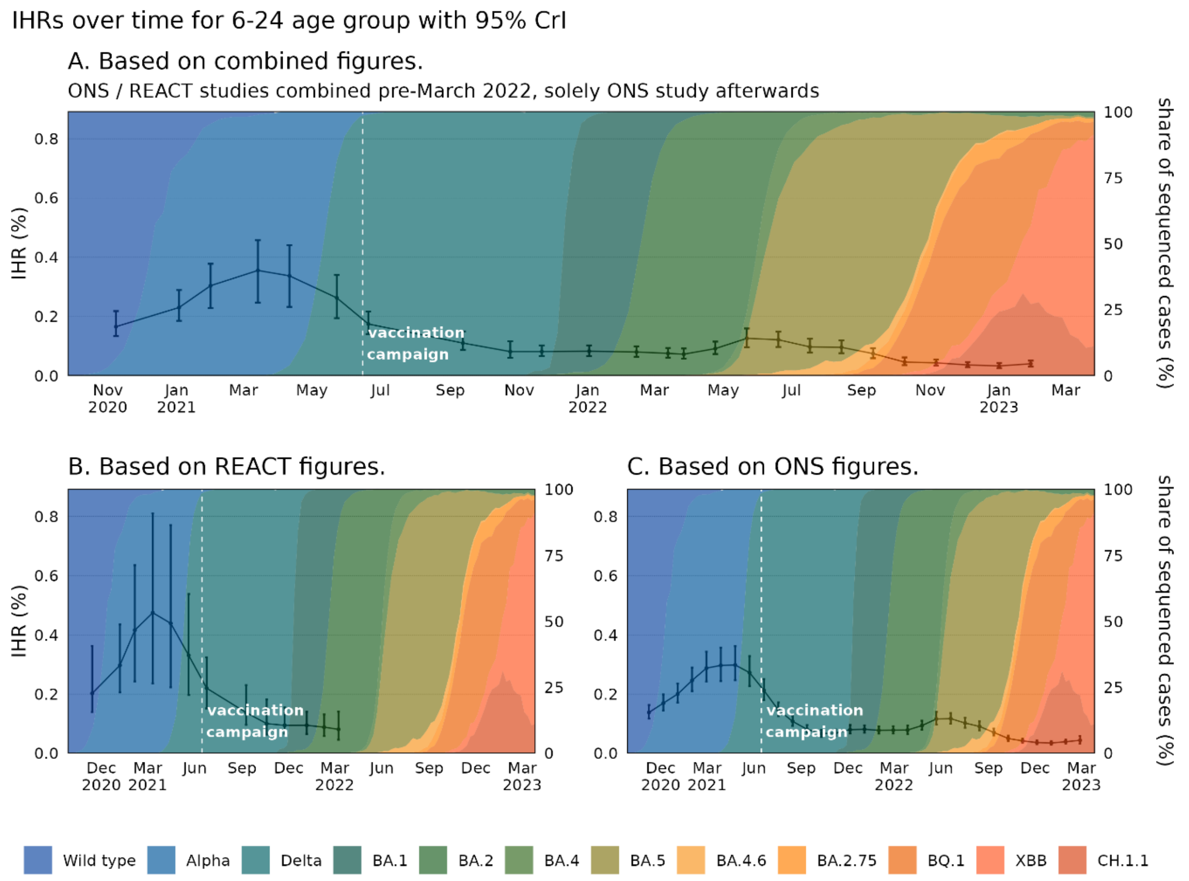


*Supplementary Figure 14 The Infection Hospitalisation Risk for the 6-24 Age Group. A: The posterior estimates of the median infection hospitalisation risk for the 6-24 age group, based on the combined REACT and ONS sampling, with 95% credible intervals. B: Posterior estimates of the median infection hospitalisation risk for the 6-24 age group, based on REACT sampling, with 95% credible intervals. C: Posterior estimates of the median infection hospitalisation risk for the 6-24 age group, based on ONS sampling, with 95% credible intervals. Not all estimates derived from the ONS CIS study have been plotted.*

## Supplementary Figure 15

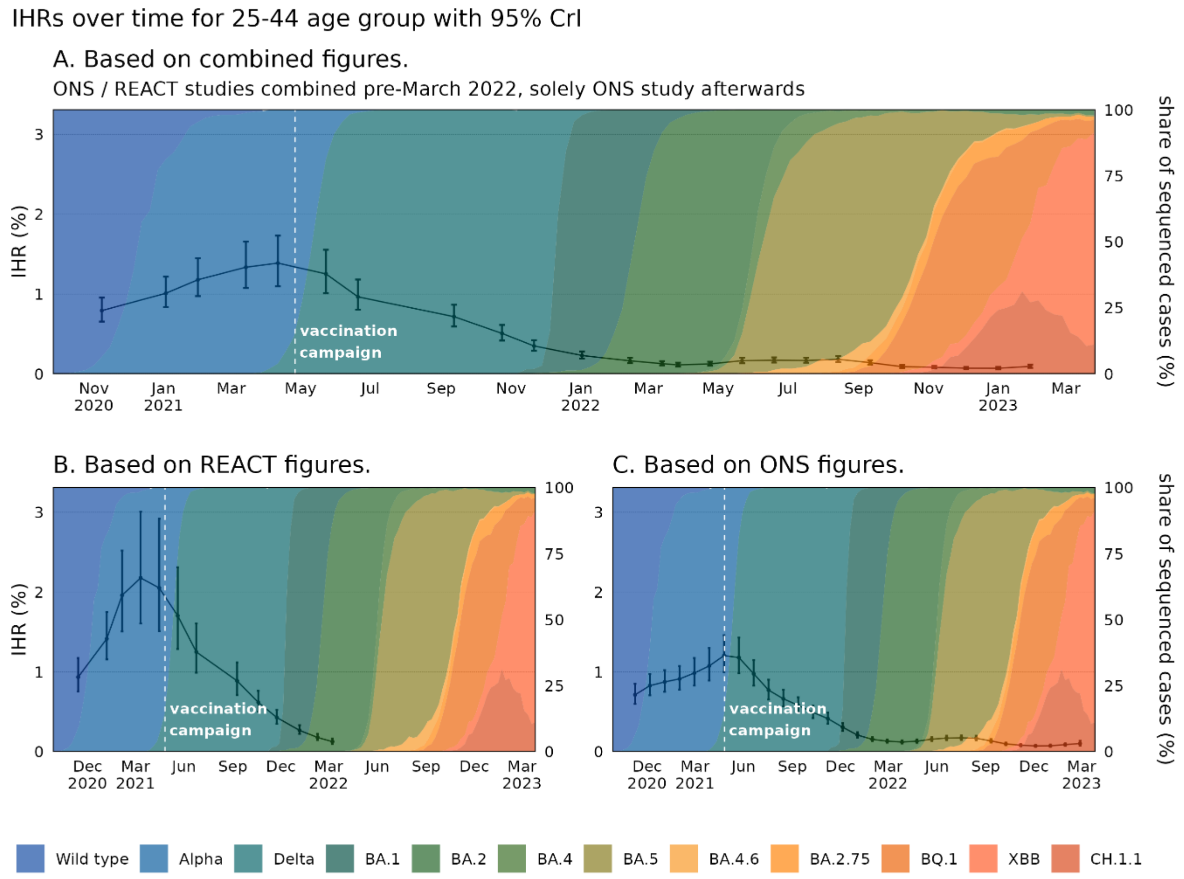


*Supplementary Figure 15 Top Panel: The Infection Hospitalisation Risk for the 25-44 Age Group. A: The posterior estimates of the median infection hospitalisation risk for the 25-44 age group, based on the combined REACT and ONS sampling, with 95% credible intervals. B: Posterior estimates of the median infection hospitalisation risk for the 25-44 age group, based on REACT sampling, with 95% credible intervals. C: Posterior estimates of the median infection hospitalisation risk for the 25-44 age group, based on ONS sampling, with 95% credible intervals. Not all estimates derived from the ONS CIS study have been plotted.*

**Supplementary Figure 16**

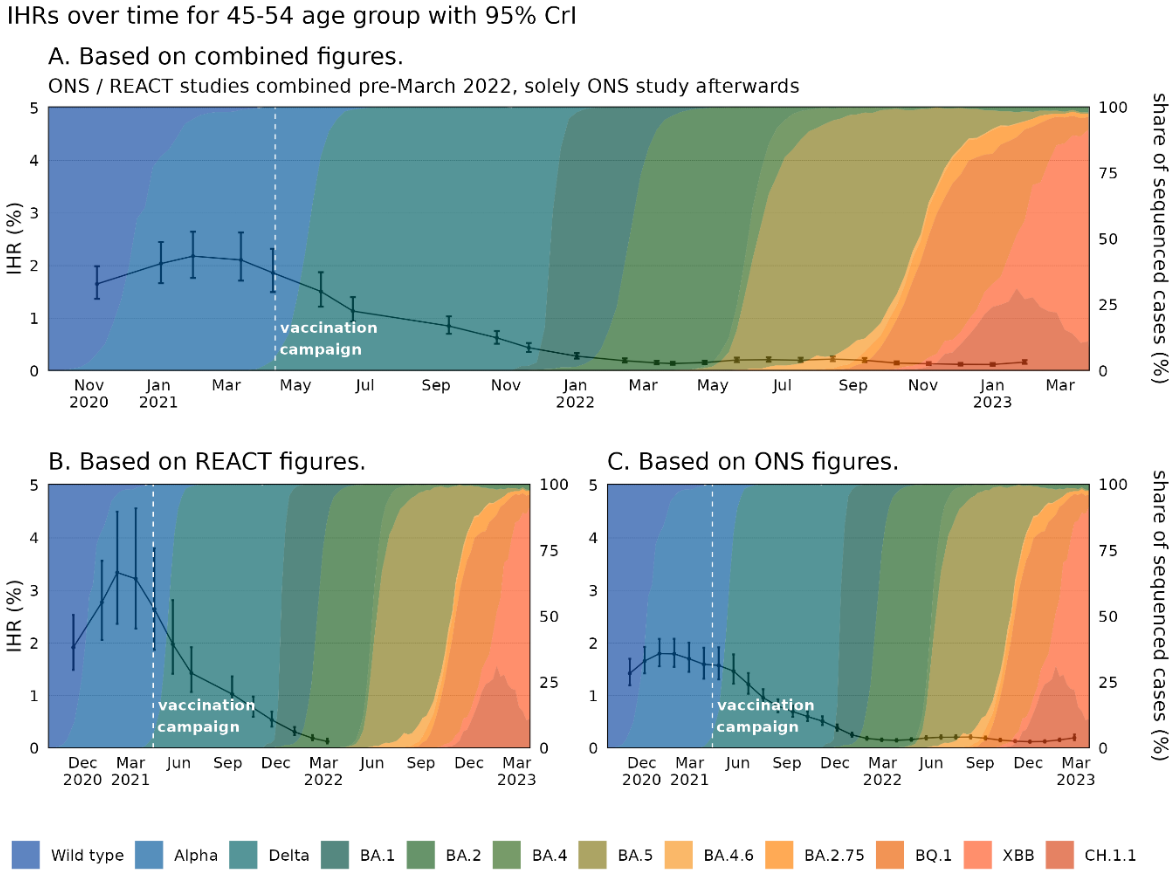


*Supplementary Figure 16 Top Panel: The Infection Hospitalisation Risk for the 45-54 Age Group. A: The posterior estimates of the median infection hospitalisation risk for the 45-54 age group, based on the combined REACT and ONS sampling, with 95% credible intervals. B: Posterior estimates of the median infection hospitalisation risk for the 45-54 age group, based on REACT sampling, with 95% credible intervals. C: Posterior estimates of the median infection hospitalisation risk for the 45-54 age group, based on ONS sampling, with 95% credible intervals. Not all estimates derived from the ONS CIS study have been plotted.*

## Supplementary Figure 17

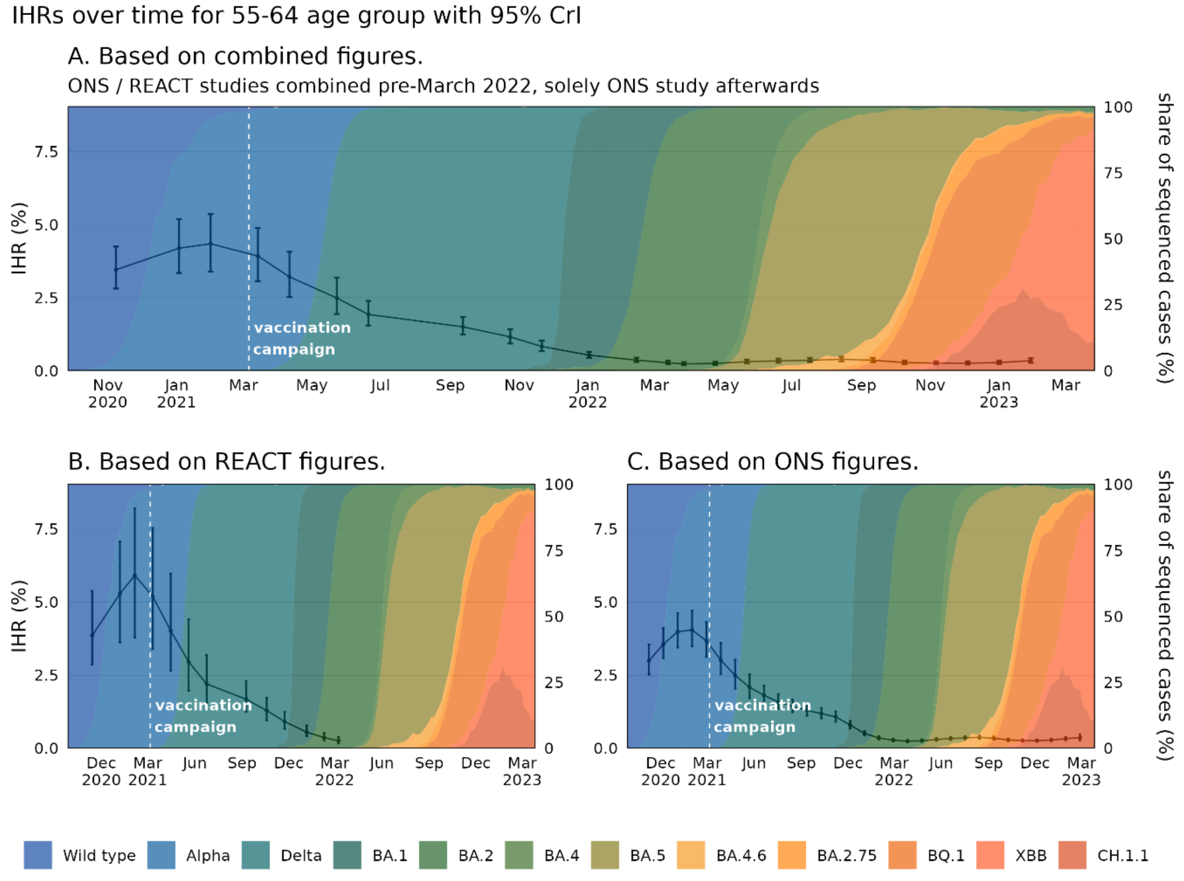


*Supplementary Figure 17 The Infection Hospitalisation Risk for the 55-64 Age Group. A: The posterior estimates of the median infection hospitalisation risk for the 55-64 age group, based on the combined REACT and ONS sampling, with 95% credible intervals. B: Posterior estimates of the median infection hospitalisation risk for the 55-64 age group, based on REACT sampling, with 95% credible intervals. C: Posterior estimates of the median infection hospitalisation risk for the 55-64 age group, based on ONS sampling, with 95% credible intervals. Not all estimates derived from the ONS CIS study have been plotted.*

## Supplementary Figure 18

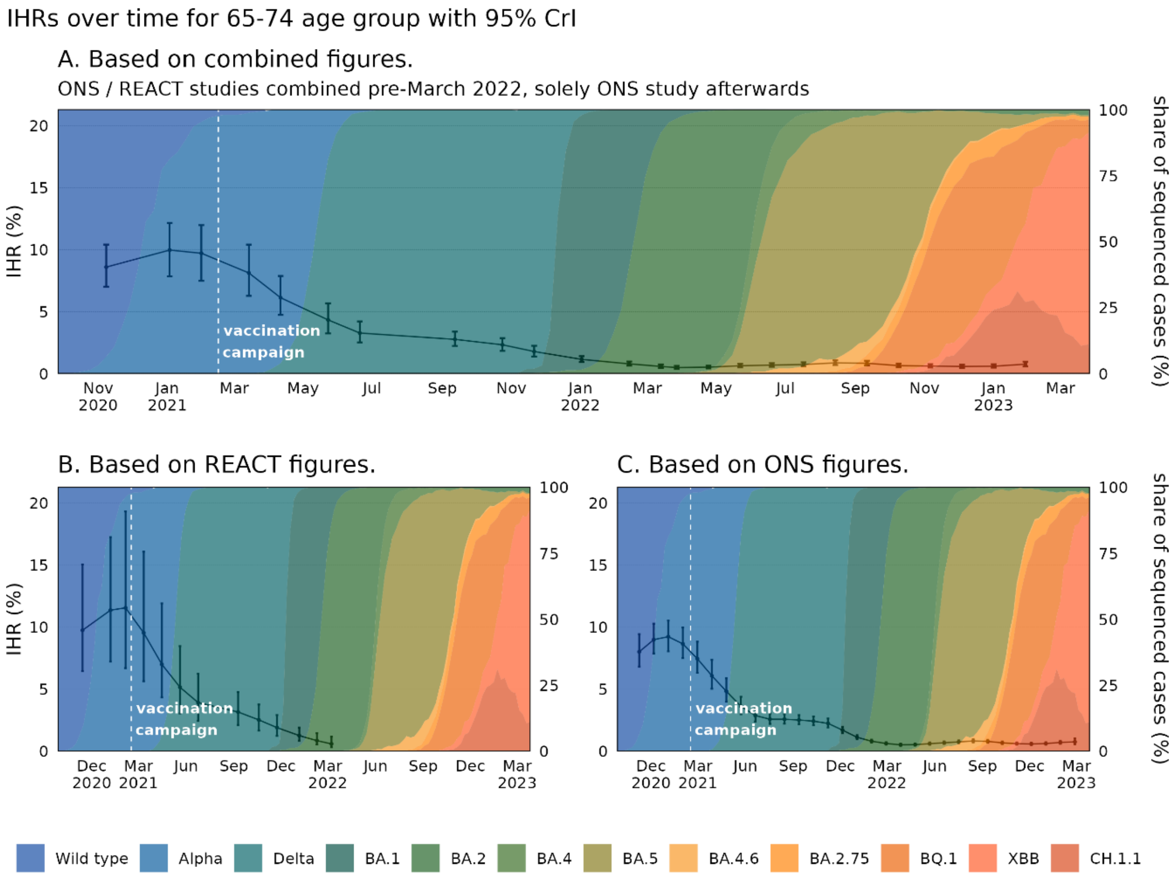


*Supplementary Figure 18 The Infection Hospitalisation Risk for the 65-74 Age Group. A: The posterior estimates of the median infection hospitalisation risk for the 65-74 age group, based on the combined REACT and ONS sampling, with 95% credible intervals. B: Posterior estimates of the median infection hospitalisation risk for the 65-74 age group, based on REACT sampling, with 95% credible intervals. C: Posterior estimates of the median infection hospitalisation risk for the 65-74 age group, based on ONS sampling, with 95% credible intervals. Not all estimates derived from the ONS CIS study have been plotted.*

## Supplementary Figure 19

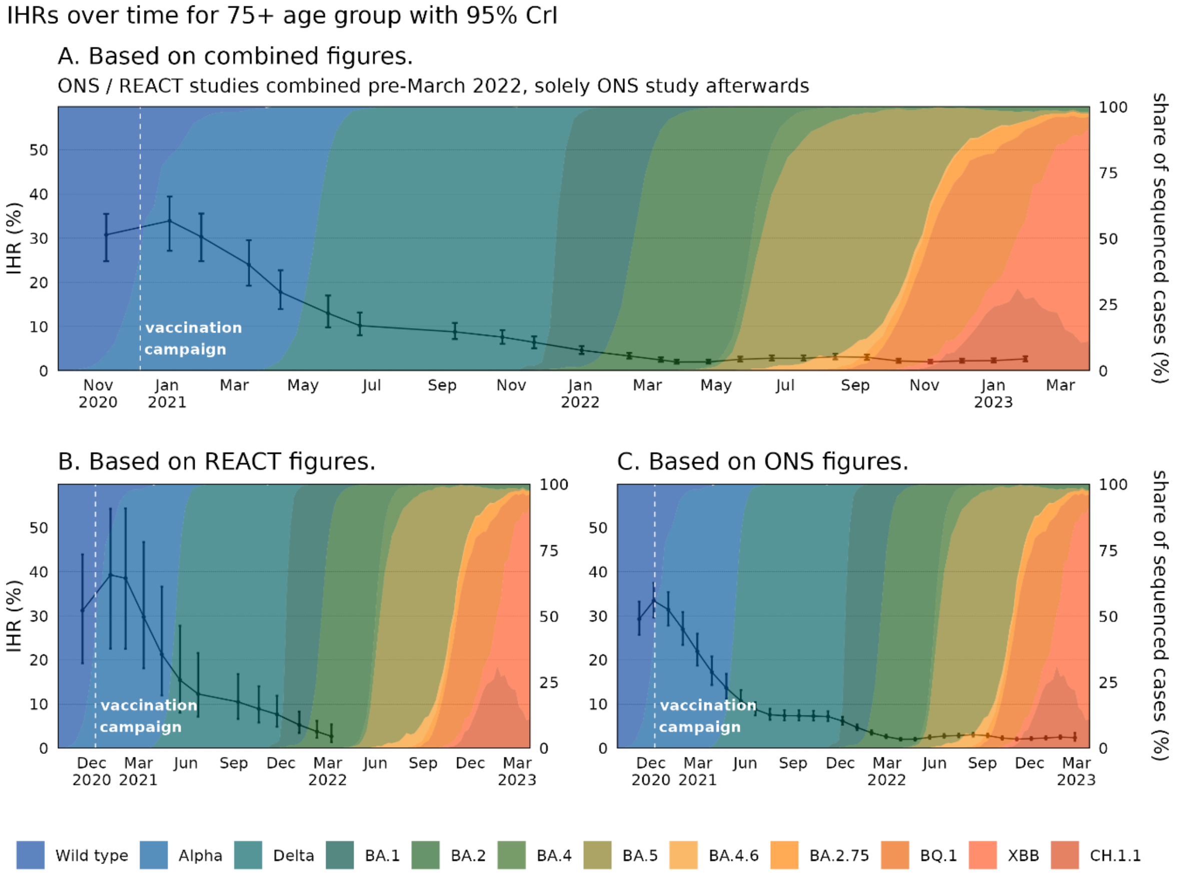


*Supplementary Figure 19 The Infection Hospitalisation Risk for the 75 and over Age Group. A: The posterior estimates of the median infection hospitalisation risk for the 75 and over age group, based on the combined REACT and ONS sampling, with 95% credible intervals. B: Posterior estimates of the median infection hospitalisation risk for the 75 and over age group, based on REACT sampling, with 95% credible intervals. C: Posterior estimates of the median infection hospitalisation risk for the 75 and over age group, based on ONS sampling, with 95% credible intervals. Not all estimates derived from the ONS CIS study have been plotted.*

## Supplementary Figure 20

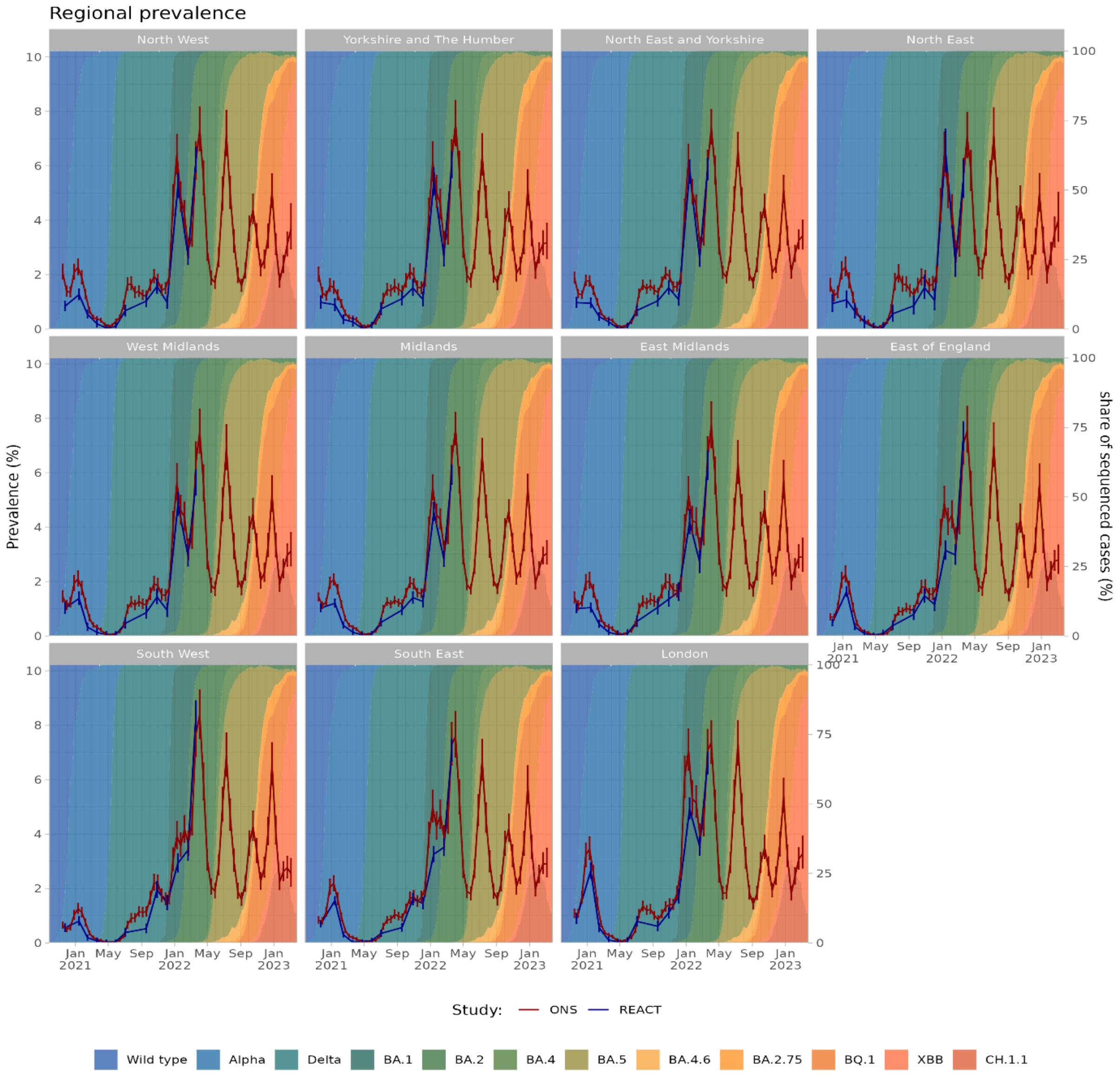


*Supplementary Figure 20 Prevalence estimates derived from the Office of National Statistics Coronavirus Infection Survey and the REACT 1 antigen survey run by Imperial College London in conjunction with Ipsos MORI for regions in England from November 2020 until March 2023 (the REACT study concluded in March 2022).*

**Supplementary Figure 21**

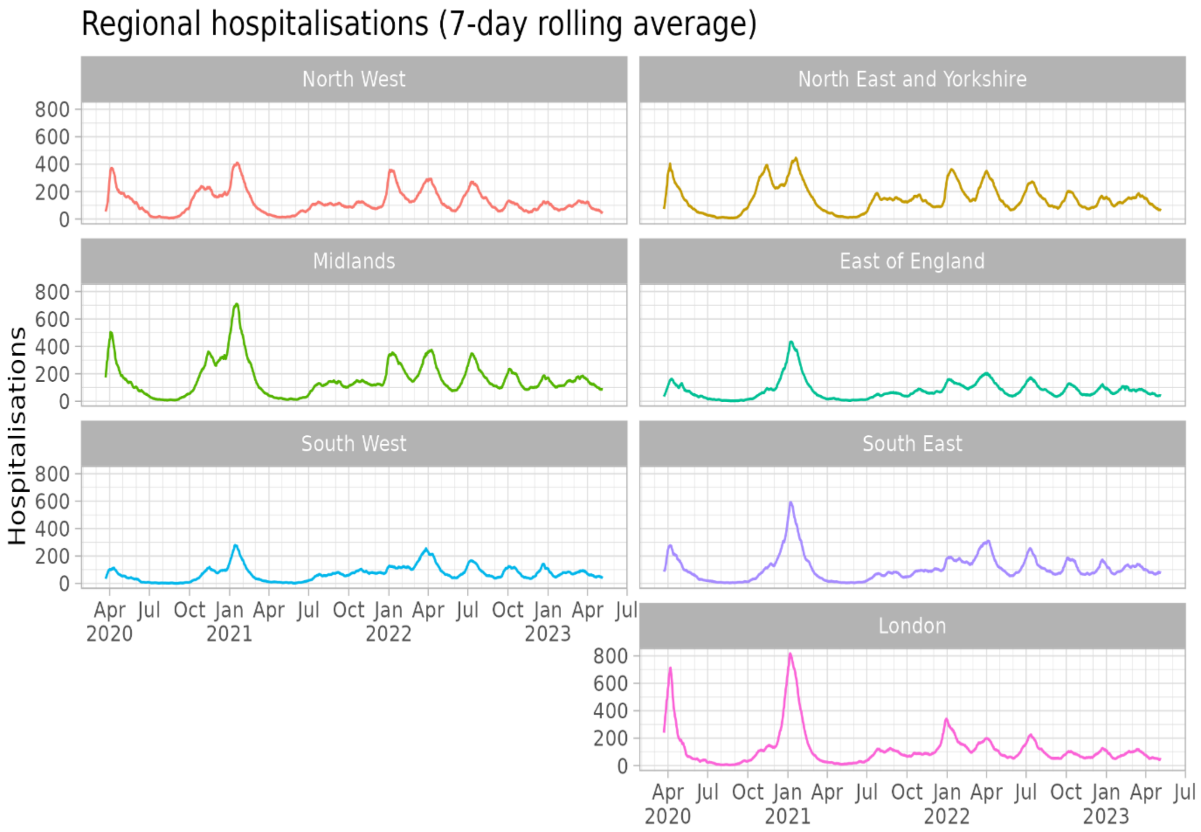


*Supplementary Figure 21 Confirmed RT-PCR positive hospital admissions by region in England from March 2020 until April 2023, sourced from NHSE&I situational reports.*

## Supplementary Figure 22

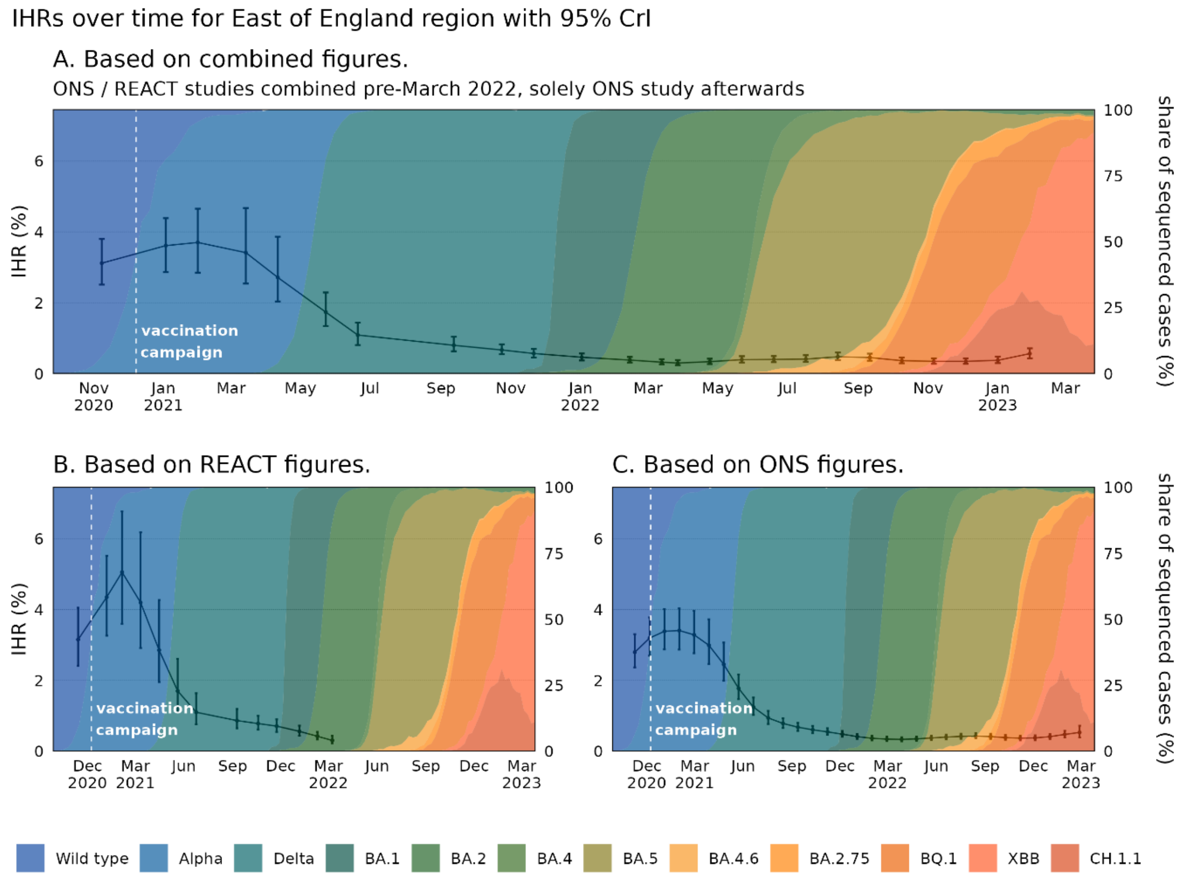


*Supplementary Figure 22 The Infection Hospitalisation Risk for the East of England. A: The posterior estimates of the median infection hospitalisation risk for the East of England, based on the combined REACT and ONS sampling, with 95% credible intervals. B: Posterior estimates of the median infection hospitalisation risk for the East of England, based on REACT sampling, with 95% credible intervals. C: Posterior estimates of the median infection hospitalisation risk for the East of England, based on ONS sampling, with 95% credible intervals. Not all estimates derived from the ONS CIS study have been plotted.*

## Supplementary Figure 23

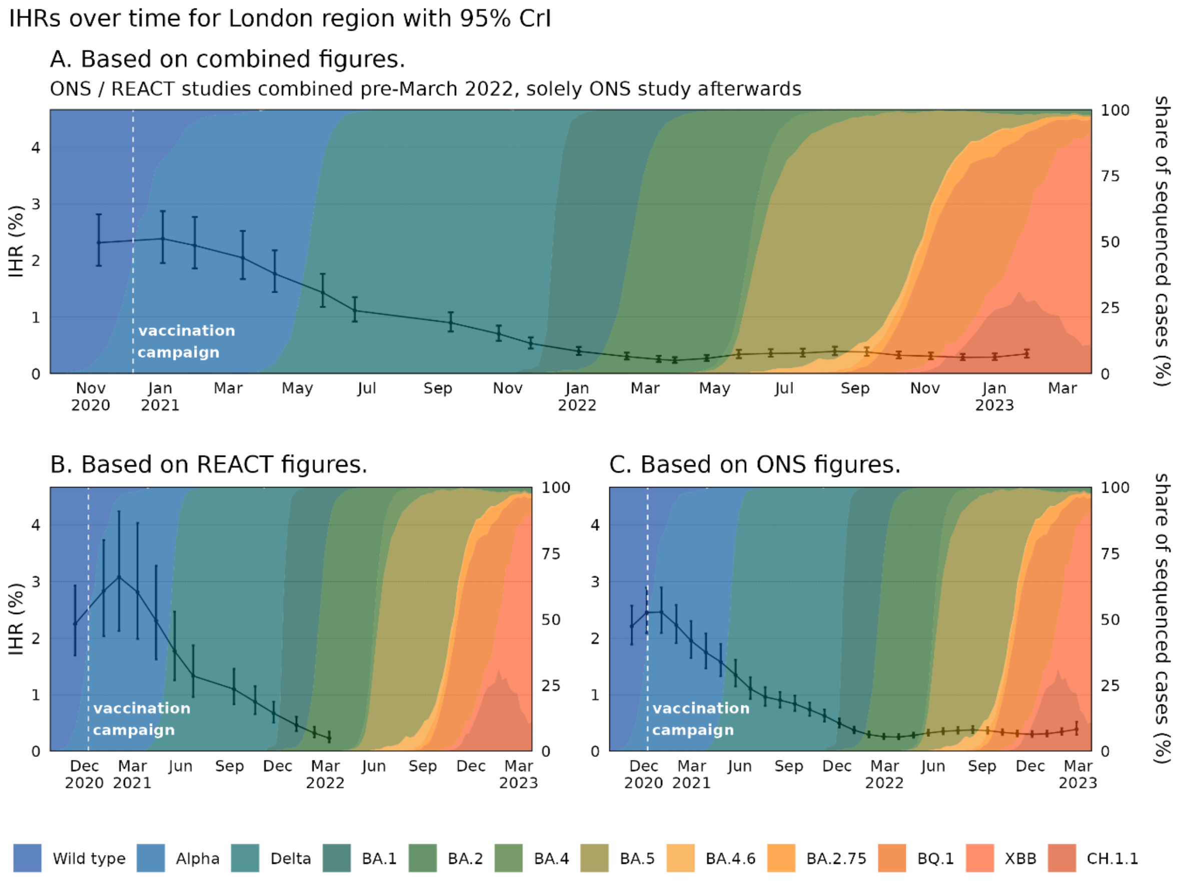


*Supplementary Figure 23 The Infection Hospitalisation Risk for London. A: The posterior estimates of the median infection hospitalisation risk for London, based on the combined REACT and ONS sampling, with 95% credible intervals. B: Posterior estimates of the median infection hospitalisation risk for London, based on REACT sampling, with 95% credible intervals. C: Posterior estimates of the median infection hospitalisation risk for London, based on ONS sampling, with 95% credible intervals. Not all estimates derived from the ONS CIS study have been plotted.*

## Supplementary Figure 24

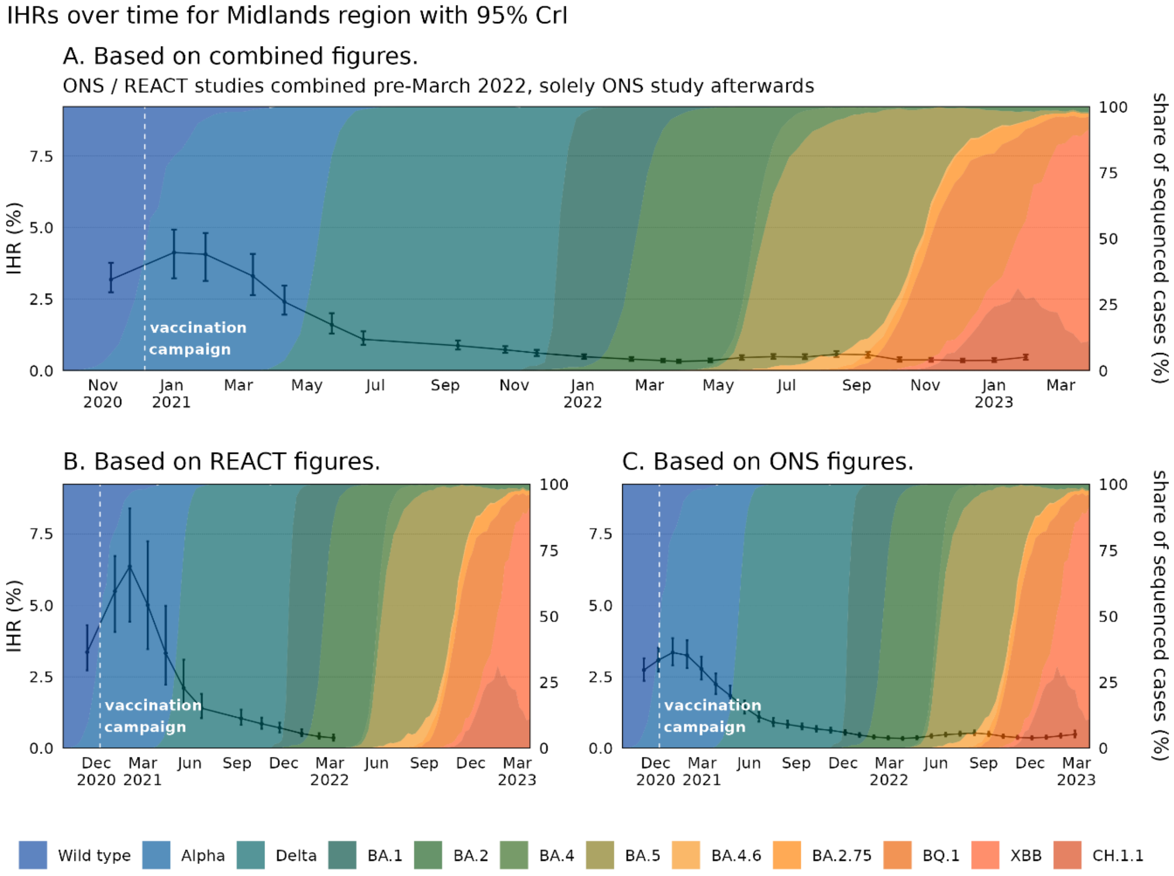


*Supplementary Figure 24 The Infection Hospitalisation Risk for the Midlands. A: The posterior estimates of the median infection hospitalisation risk for the Midlands, based on the combined REACT and ONS sampling, with 95% credible intervals. B: Posterior estimates of the median infection hospitalisation risk for the Midlands, based on REACT sampling, with 95% credible intervals. C: Posterior estimates of the median infection hospitalisation risk for the Midlands, based on ONS sampling, with 95% credible intervals. Not all estimates derived from the ONS CIS study have been plotted.*

## Supplementary Figure 25

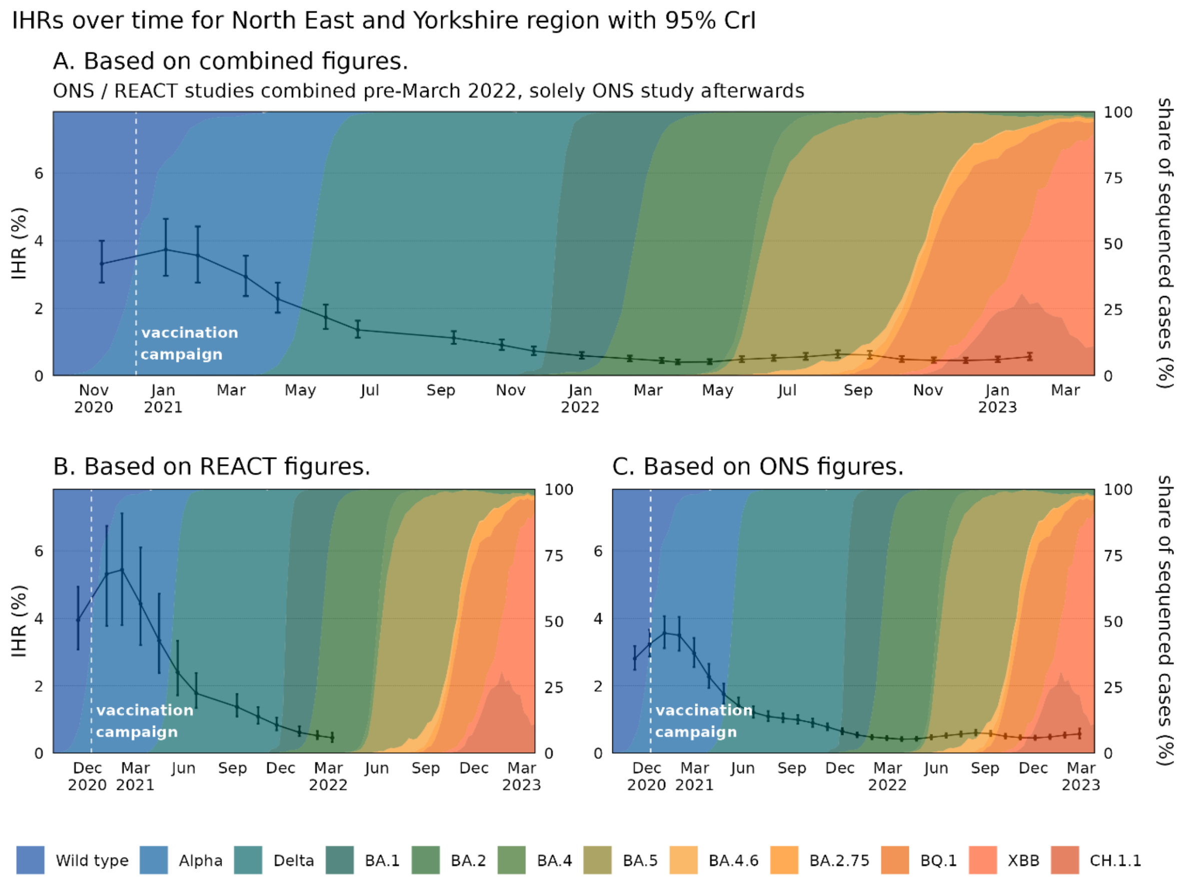


*Supplementary Figure 25 The Infection Hospitalisation Risk for the North East and Yorkshire. A: The posterior estimates of the median infection hospitalisation risk for the North East and Yorkshire, based on the combined REACT and ONS sampling, with 95% credible intervals. B: Posterior estimates of the median infection hospitalisation risk for the North East and Yorkshire, based on REACT sampling, with 95% credible intervals. C: Posterior estimates of the median infection hospitalisation risk for the North East and Yorkshire, based on ONS sampling, with 95% credible intervals. Not all estimates derived from the ONS CIS study have been plotted.*

## Supplementary Figure 26

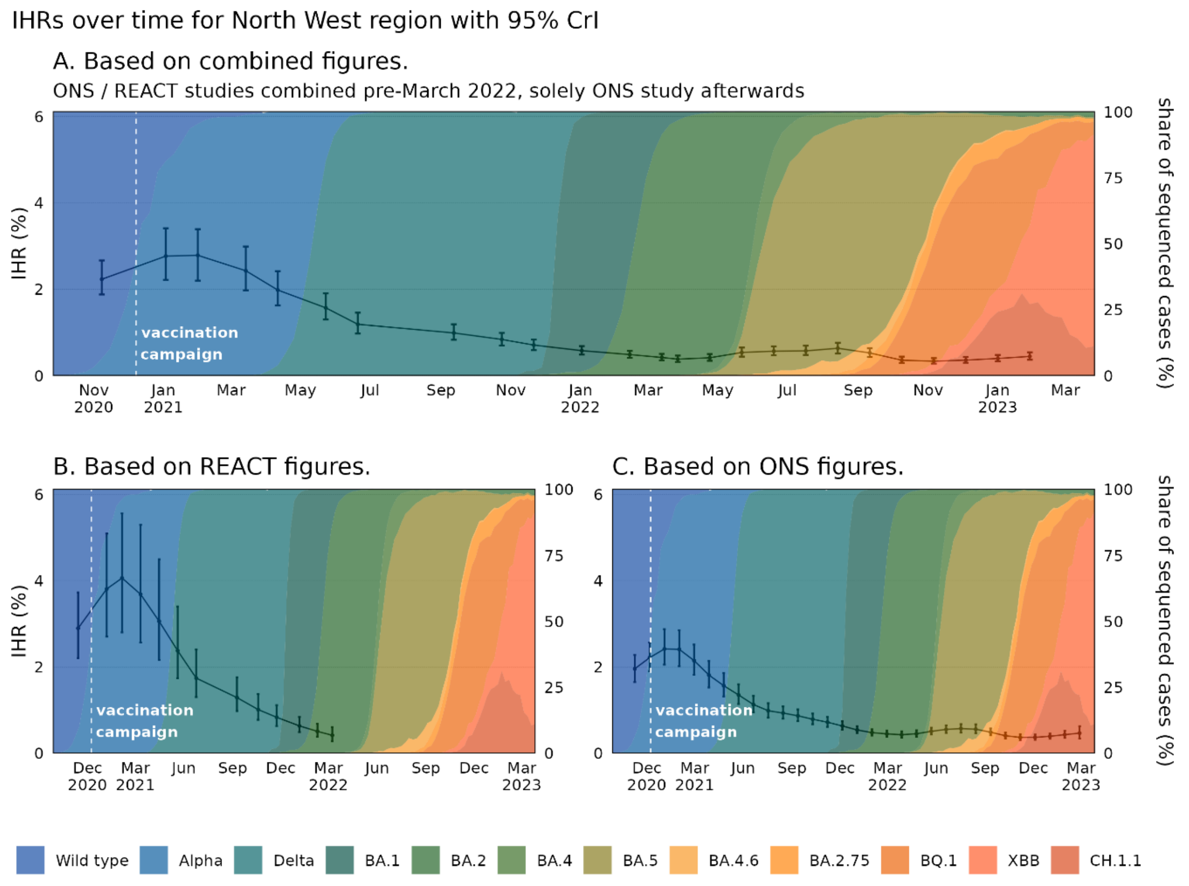


*Supplementary Figure 26 The Infection Hospitalisation Risk for the North West. A: The posterior estimates of the median infection hospitalisation risk for the North West, based on the combined REACT and ONS sampling, with 95% credible intervals. B: Posterior estimates of the median infection hospitalisation risk for the North West, based on REACT sampling, with 95% credible intervals. C: Posterior estimates of the median infection hospitalisation risk for the North West, based on ONS sampling, with 95% credible intervals. Not all estimates derived from the ONS CIS study have been plotted.*

**Supplementary Figure 27**

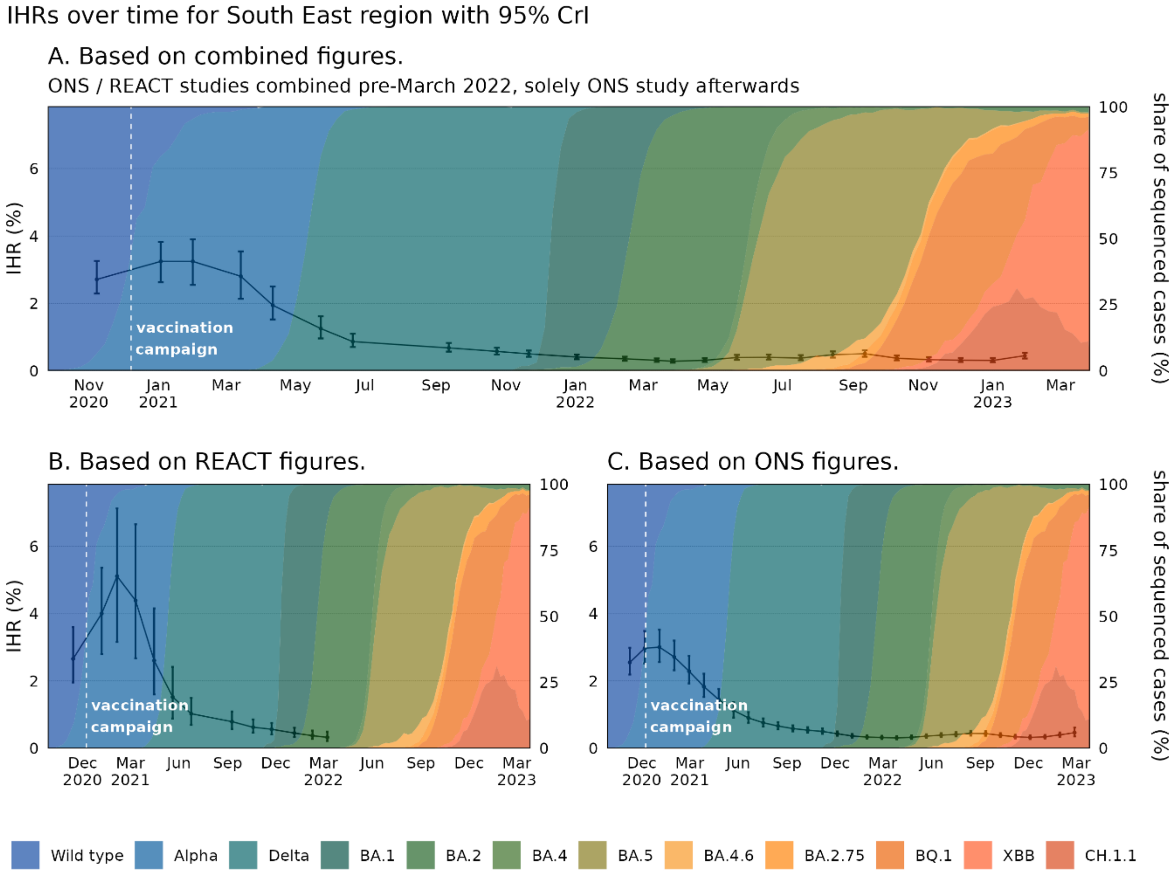


*Supplementary Figure 27 The Infection Hospitalisation Risk for the South East. A: The posterior estimates of the median infection hospitalisation risk for the South East, based on the combined REACT and ONS sampling, with 95% credible intervals. B: Posterior estimates of the median infection hospitalisation risk for the South East, based on REACT sampling, with 95% credible intervals. C: Posterior estimates of the median infection hospitalisation risk for the South East, based on ONS sampling, with 95% credible intervals. Not all estimates derived from the ONS CIS study have been plotted.*

.

## Supplementary Figure 28

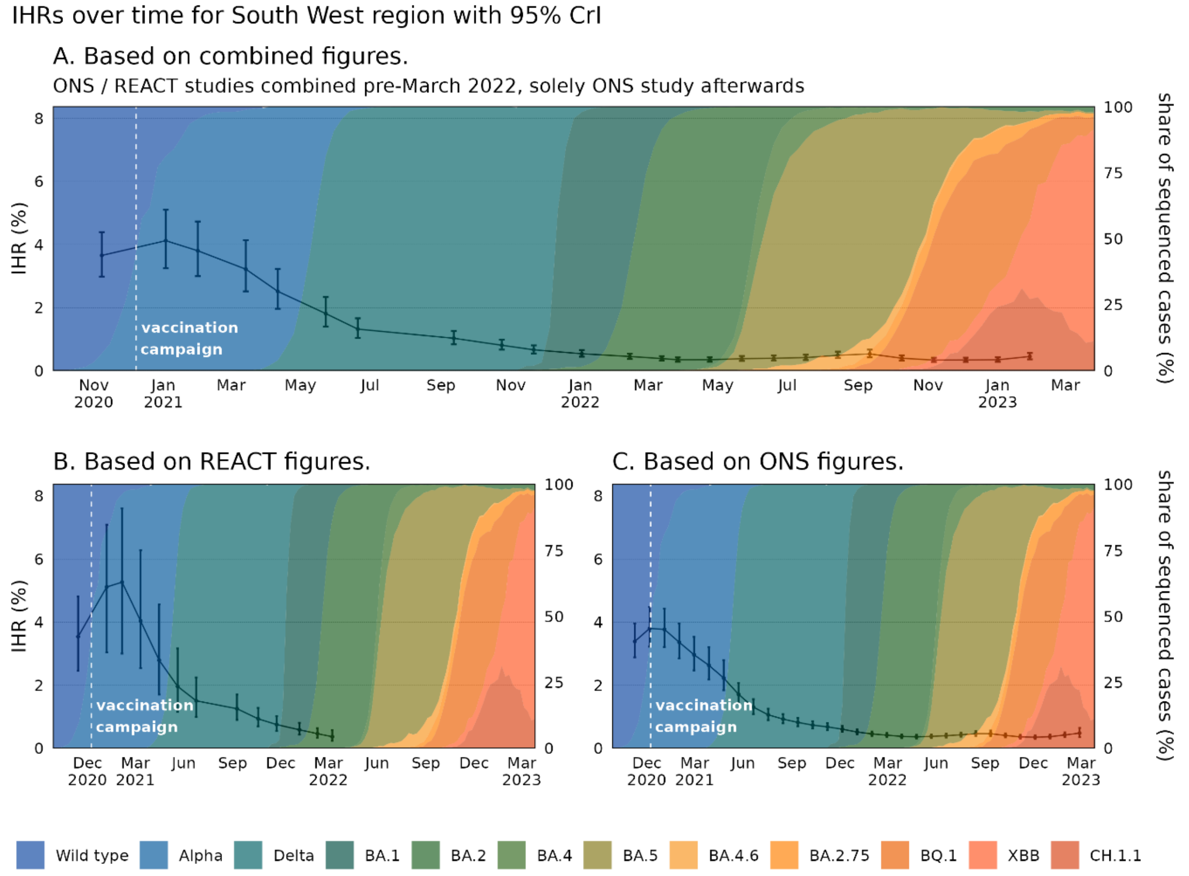


*Supplementary Figure 28 The Infection Hospitalisation Risk for the South West. A: The posterior estimates of the median infection hospitalisation risk for the South West, based on the combined REACT and ONS sampling, with 95% credible intervals. B: Posterior estimates of the median infection hospitalisation risk for the South West, based on REACT sampling, with 95% credible intervals. C: Posterior estimates of the median infection hospitalisation risk for the South West, based on ONS sampling, with 95% credible intervals. Not all estimates derived from the ONS CIS study have been plotted.*

**Supplementary Figure 29**

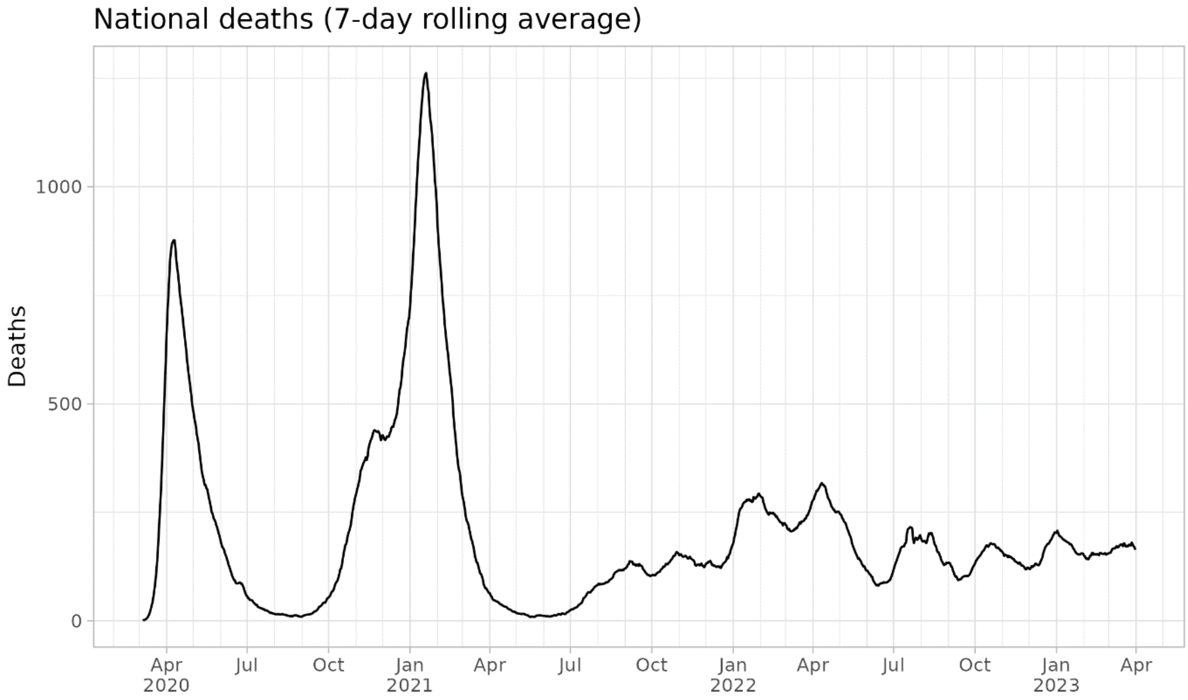


*Supplementary Figure 29 Deaths within 60 days of a confirmed RT-PCR positive test from March 2020 until May 2023, sourced from UKHSA COVID-19 death linelist.*

**Supplementary Figure 30**

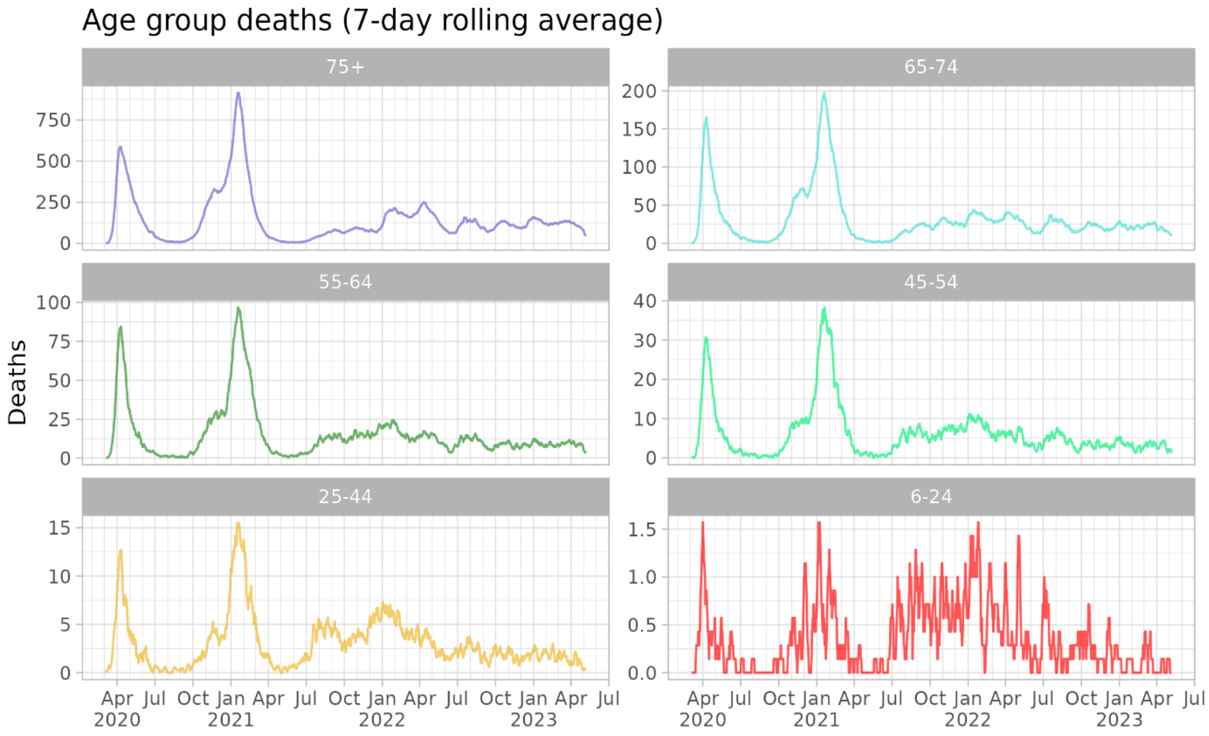


*Supplementary Figure 30 Deaths within 60 days of a confirmed RT-PCR positive test in England by age group from March 2020 until May 2023, sourced from UKHSA COVID-19 death linelist.*

## Supplementary Figure 31

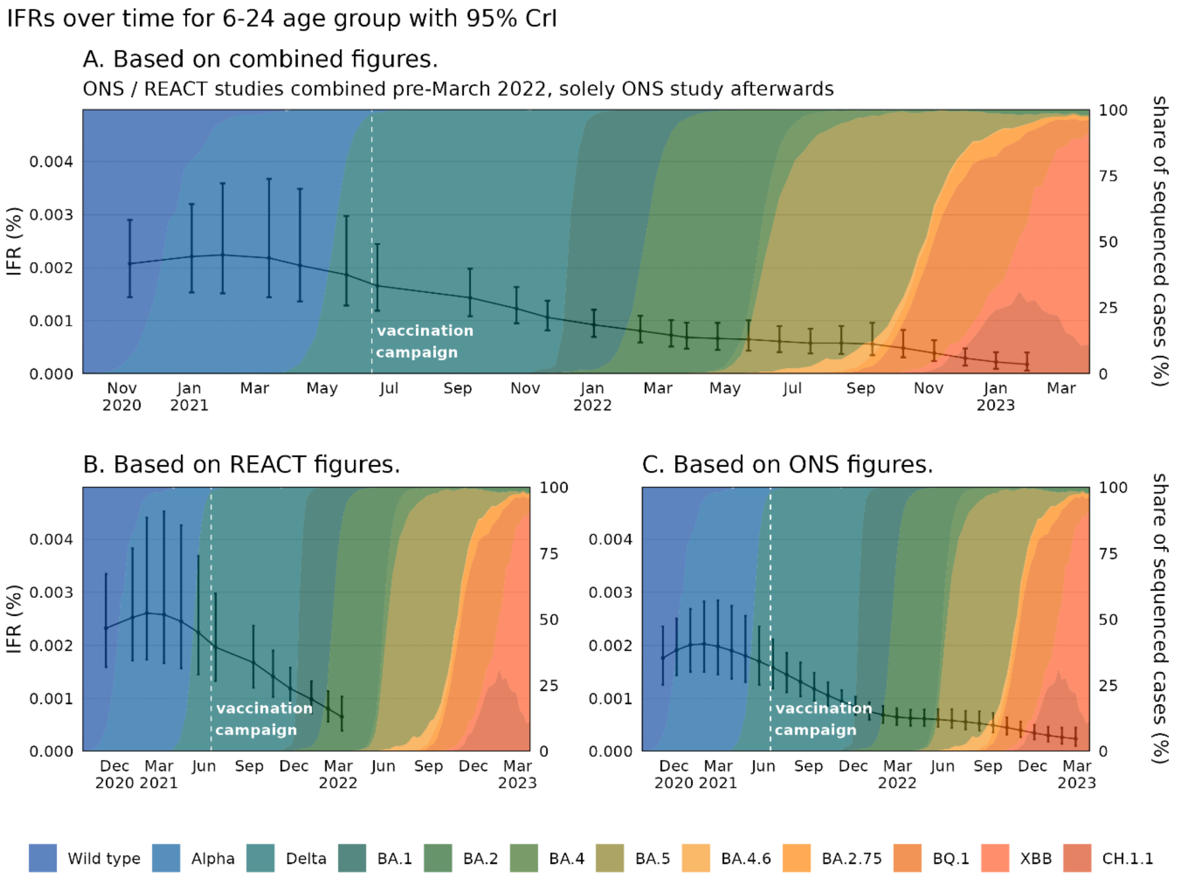


*Supplementary Figure 31 The Infection Fatality Risk for the 6-24 Age Group. A: The posterior estimates of the median infection fatality risk for the 6-24 age group, based on the combined REACT and ONS sampling, with 95% credible intervals. B: Posterior estimates of the median infection fatality risk for the 6-24 age group, based on REACT sampling, with 95% credible intervals. C: Posterior estimates of the median infection fatality risk for the 6-24 age group, based on ONS sampling, with 95% credible intervals. Not all estimates derived from the ONS CIS study have been plotted.*

## Supplementary Figure 32

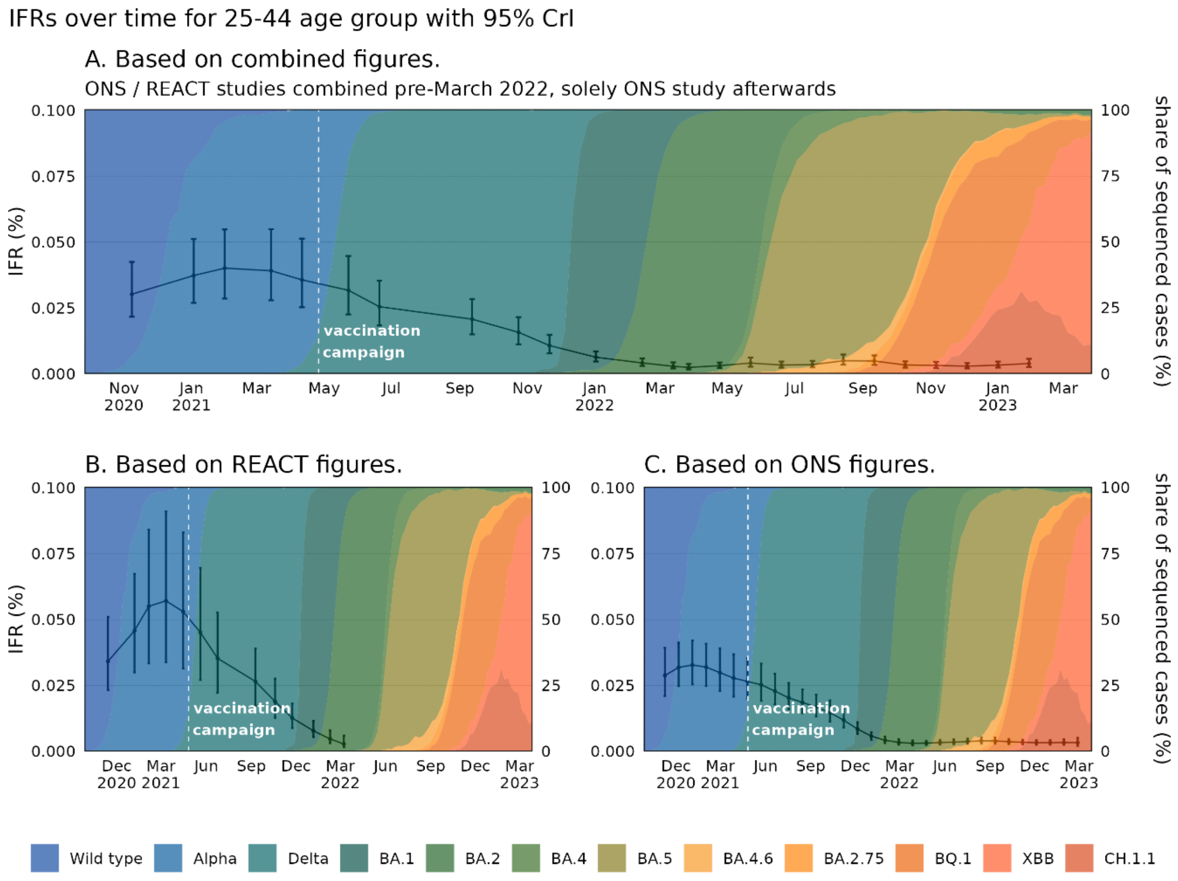


*Supplementary Figure 32 The Infection Fatality Risk for the 25-44 Age Group. A: The posterior estimates of the median infection fatality risk for the 25-44 age group, based on the combined REACT and ONS sampling, with 95% credible intervals. B: Posterior estimates of the median infection fatality risk for the 25-44 age group, based on REACT sampling, with 95% credible intervals. C: Posterior estimates of the median infection fatality risk for the 25-44 age group, based on ONS sampling, with 95% credible intervals. Not all estimates derived from the ONS CIS study have been plotted.*

## Supplementary Figure 33

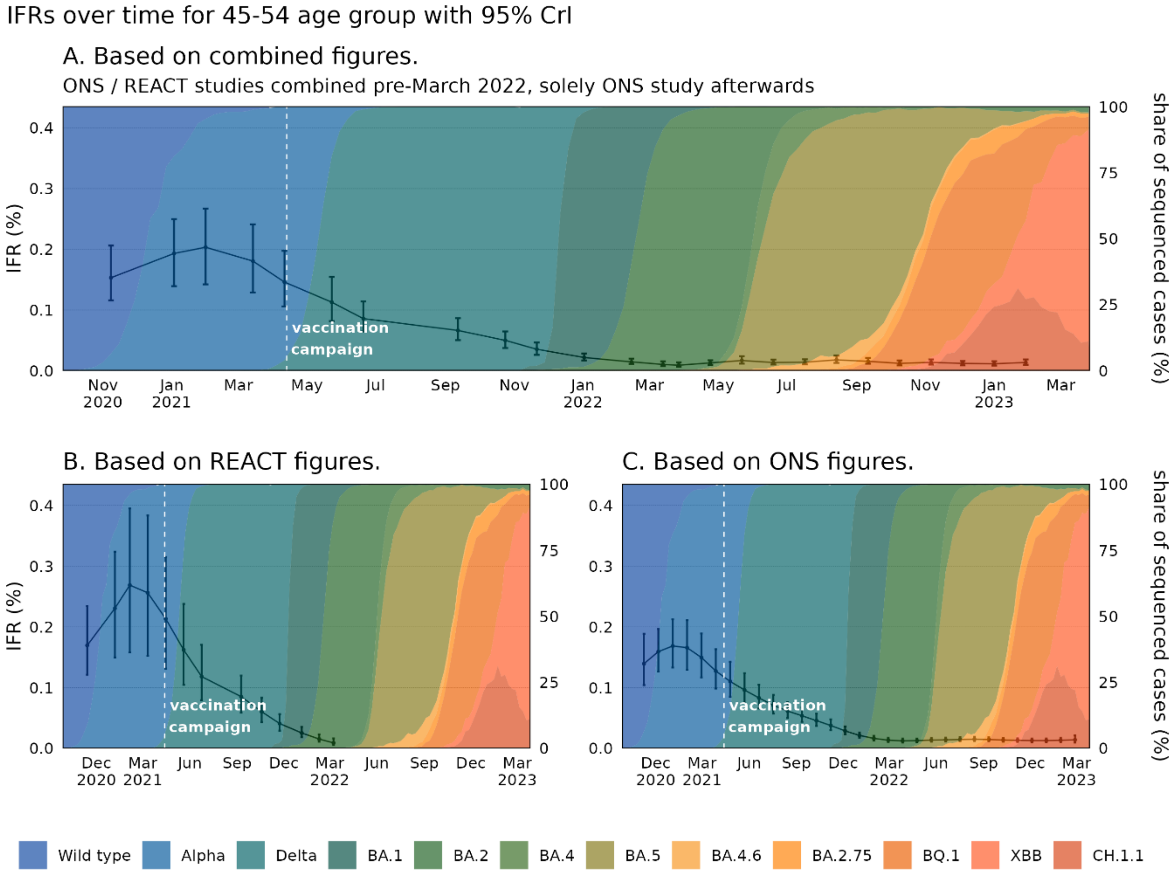


*Supplementary Figure 33 Top Panel: The Infection Fatality Risk for the 45-54 Age Group. A: The posterior estimates of the median infection fatality risk for the 45-54 age group, based on the combined REACT and ONS sampling, with 95% credible intervals. B: Posterior estimates of the median infection fatality risk for the 45-54 age group, based on REACT sampling, with 95% credible intervals. C: Posterior estimates of the median infection fatality risk for the 45-54 age group, based on ONS sampling, with 95% credible intervals. Not all estimates derived from the ONS CIS study have been plotted.*

## Supplementary Figure 34

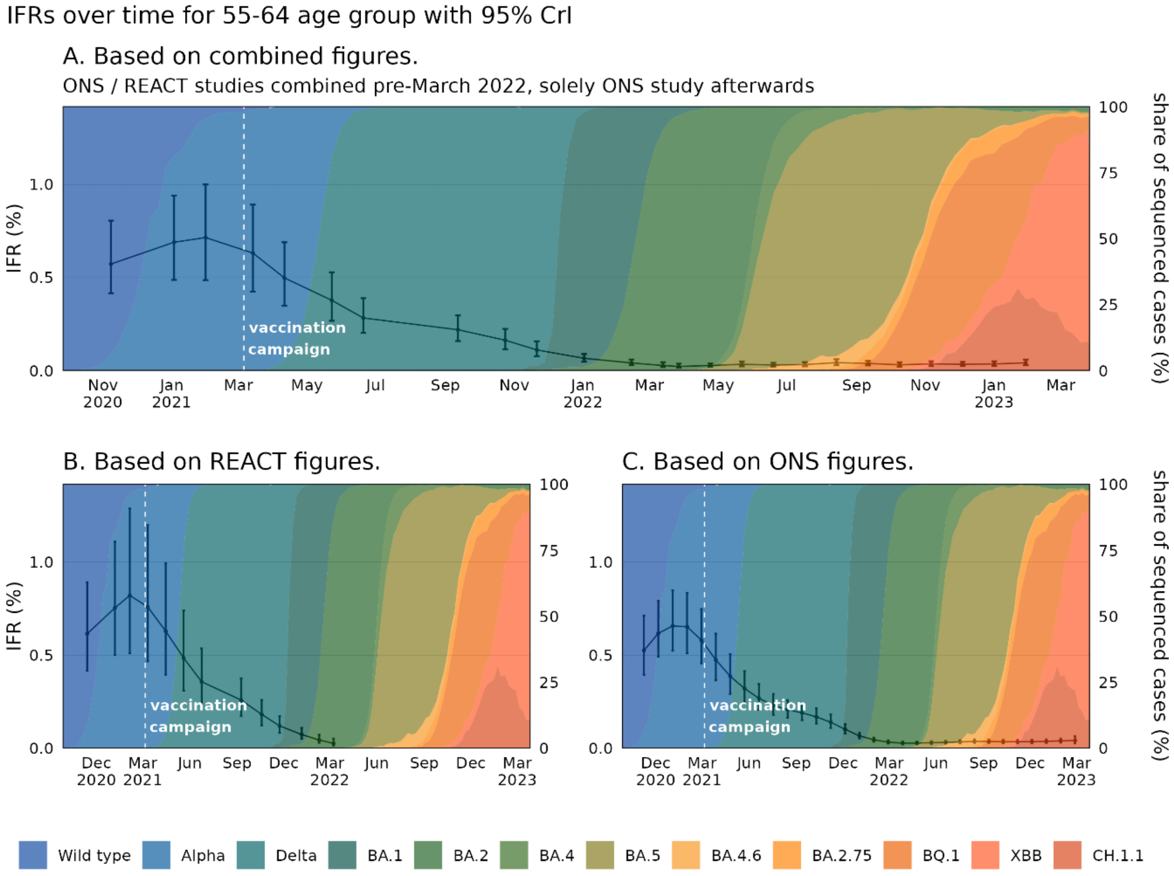


*Supplementary Figure 34 The Infection Fatality Risk for the 55-64 Age Group. A: The posterior estimates of the median infection fatality risk for the 55-64 age group, based on the combined REACT and ONS sampling, with 95% credible intervals. B: Posterior estimates of the median infection fatality risk for the 55-64 age group, based on REACT sampling, with 95% credible intervals. C: Posterior estimates of the median infection fatality risk for the 55-64 age group, based on ONS sampling, with 95% credible intervals. Not all estimates derived from the ONS CIS study have been plotted.*

**Supplementary Figure 35**

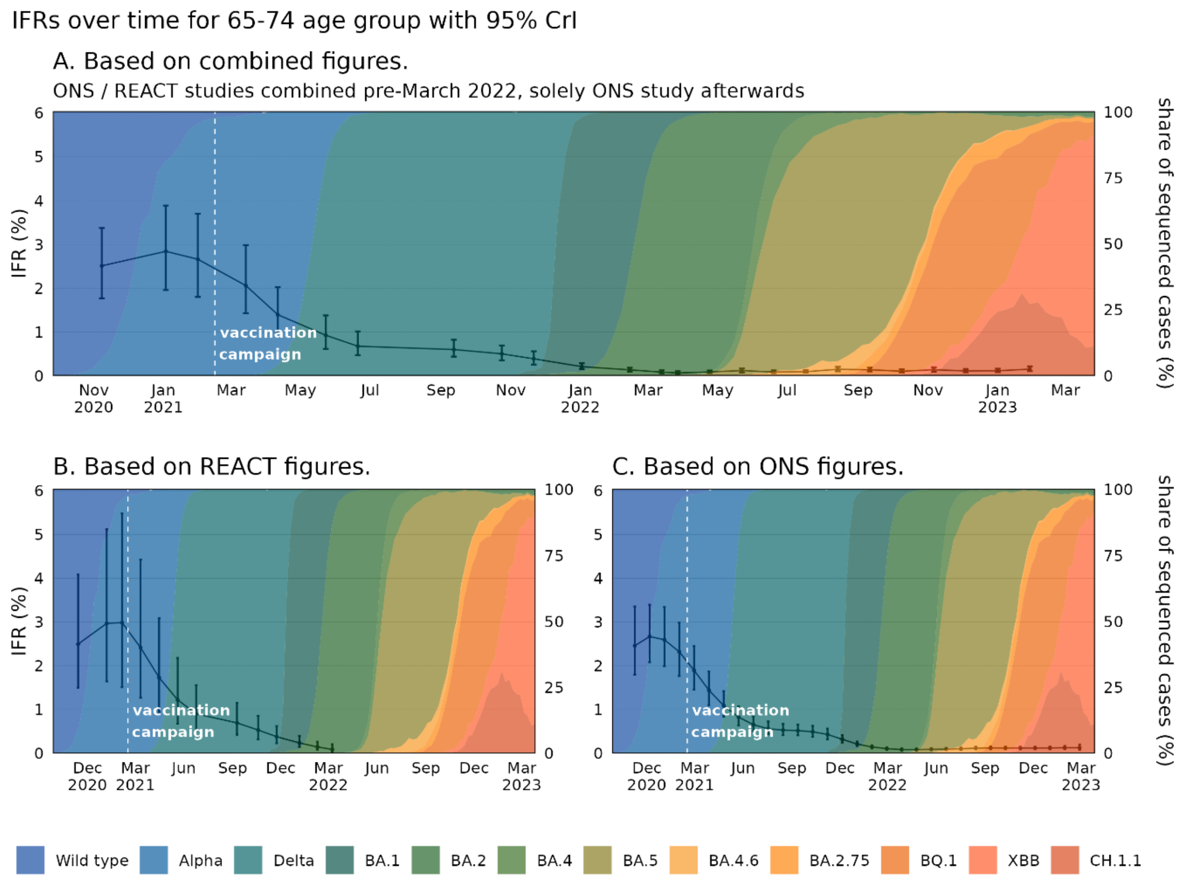


*Supplementary Figure 35 The Infection Fatality Risk for the 65-74 Age Group. A: The posterior estimates of the median infection fatality risk for the 65-74 age group, based on the combined REACT and ONS sampling, with 95% credible intervals. B: Posterior estimates of the median infection fatality risk for the 65-74 age group, based on REACT sampling, with 95% credible intervals. C: Posterior estimates of the median infection fatality risk for the 65-74 age group, based on ONS sampling, with 95% credible intervals. Not all estimates derived from the ONS CIS study have been plotted.*

**Supplementary Figure 36**

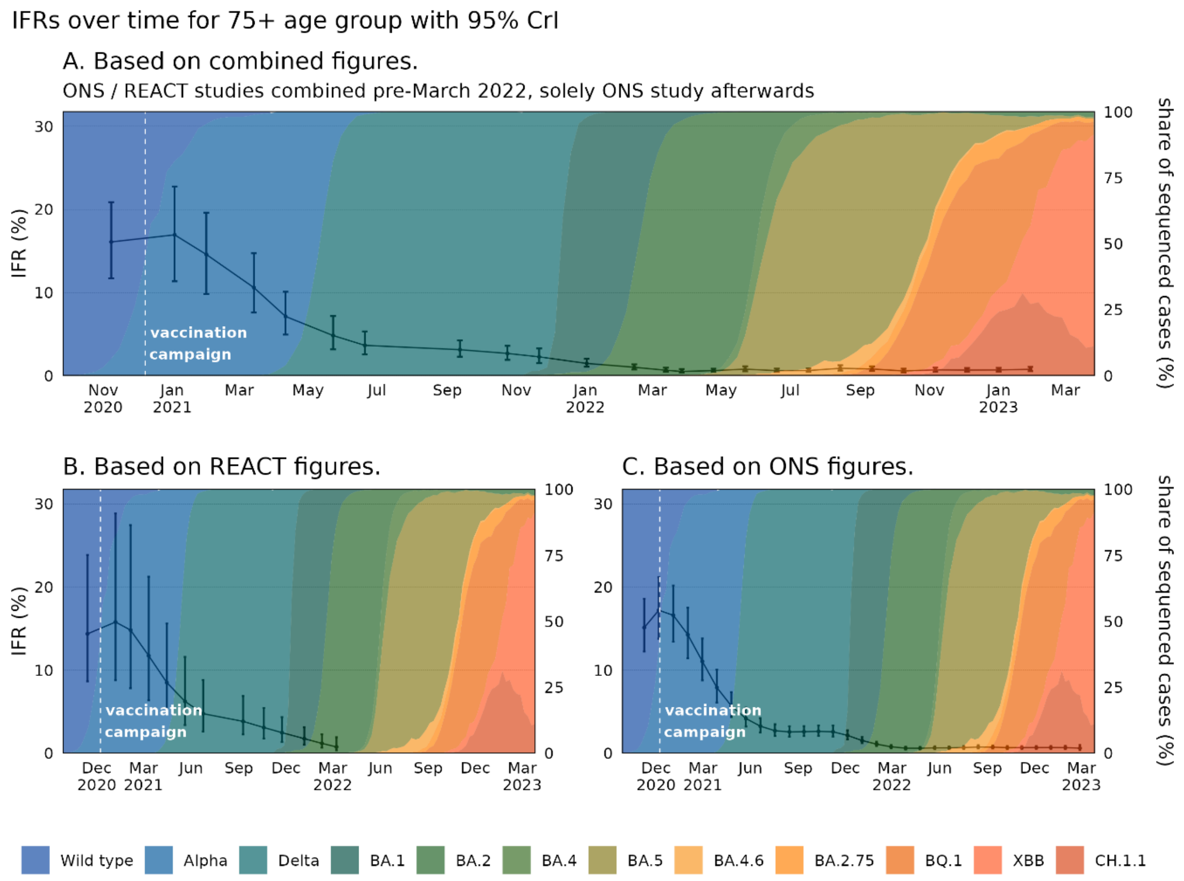


*Supplementary Figure 36 Top Panel: The Infection Fatality Risk for the 75 and over Age Group. A: The posterior estimates of the median infection fatality risk for the 75 and over age group, based on the combined REACT and ONS sampling, with 95% credible intervals. B: Posterior estimates of the median infection fatality risk for the 75 and over age group, based on REACT sampling, with 95% credible intervals. C: Posterior estimates of the median infection fatality risk for the 75 and over age group, based on ONS sampling, with 95% credible intervals. Not all estimates derived from the ONS CIS study have been plotted.*

**Supplementary Figure 37**

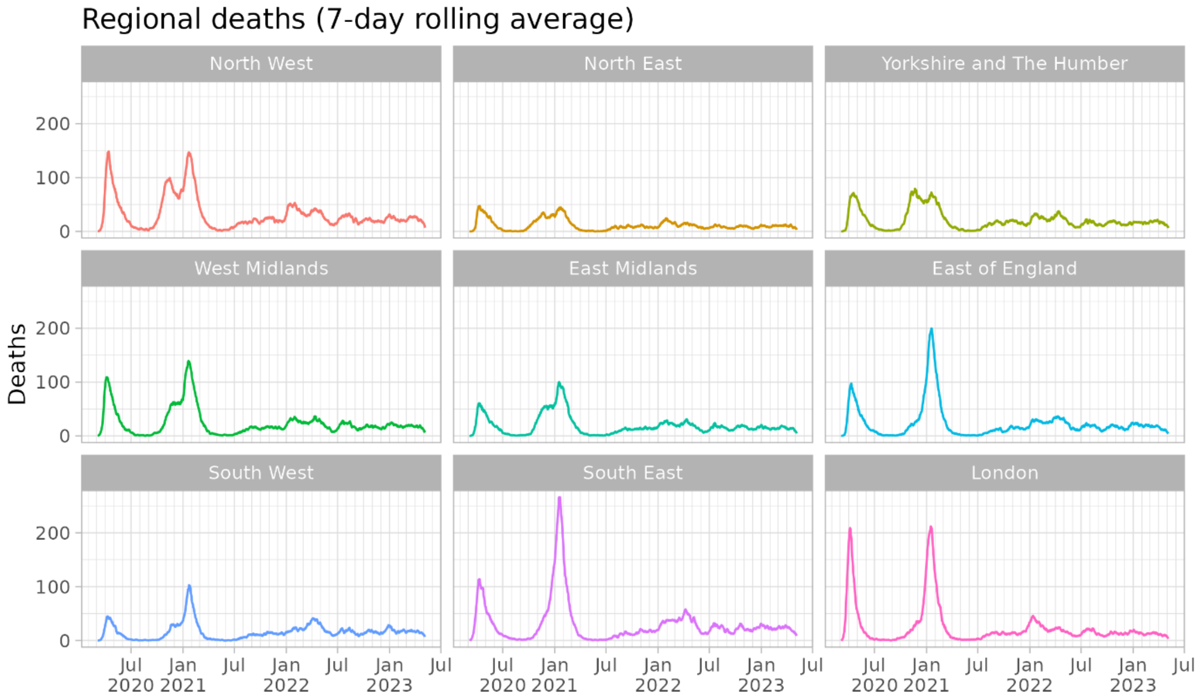


*Supplementary Figure 37 Deaths within 60 days of a confirmed RT-PCR positive test in England by age group from March 2020 until May 2023, sourced from UKHSA COVID-19 death linelist.*

## Supplementary Figure 38

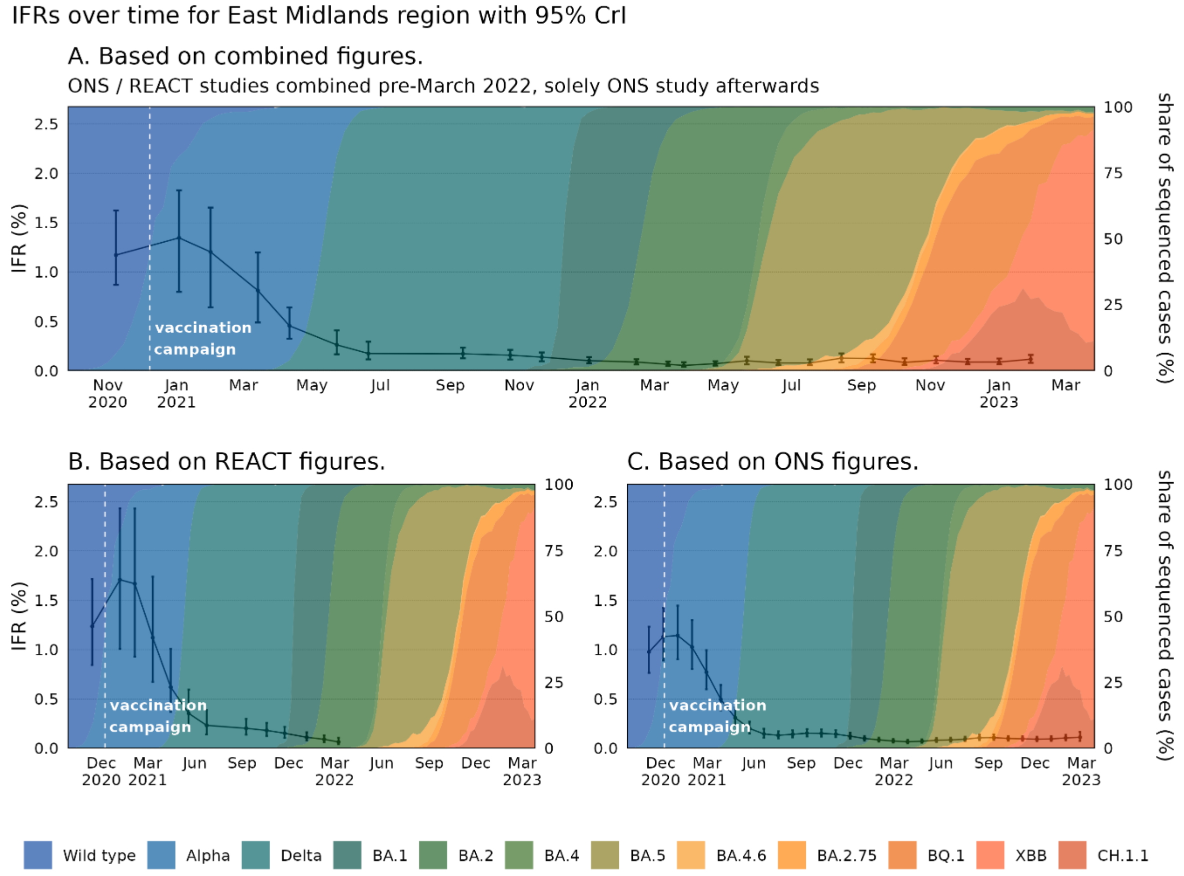


*Supplementary Figure 38 The Infection Fatality Risk for the East Midlands. A: The posterior estimates of the median infection fatality risk for the East Midlands, based on the combined REACT and ONS sampling, with 95% credible intervals. B: Posterior estimates of the median infection fatality risk for the East Midlands, based on REACT sampling, with 95% credible intervals. C: Posterior estimates of the median infection fatality risk for the East Midlands, based on ONS sampling, with 95% credible intervals. Not all estimates derived from the ONS CIS study have been plotted.*

**Supplementary Figure 39**

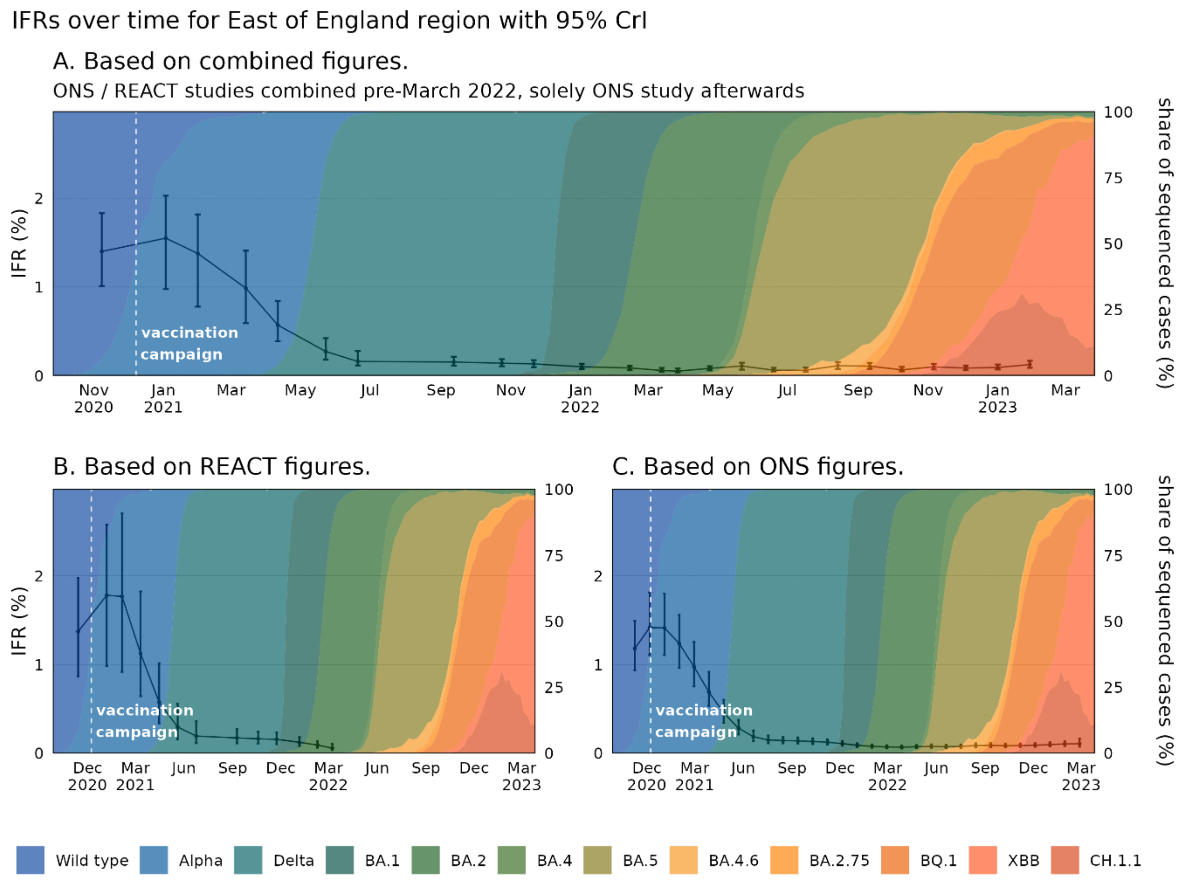


*Supplementary Figure 39 The Infection Fatality Risk for the East of England. A: The posterior estimates of the median infection fatality risk for the East of England, based on the combined REACT and ONS sampling, with 95% credible intervals. B: Posterior estimates of the median infection fatality risk for the East of England, based on REACT sampling, with 95% credible intervals. C: Posterior estimates of the median infection fatality risk for the East of England, based on ONS sampling, with 95% credible intervals. Not all estimates derived from the ONS CIS study have been plotted.*

## Supplementary Figure 40

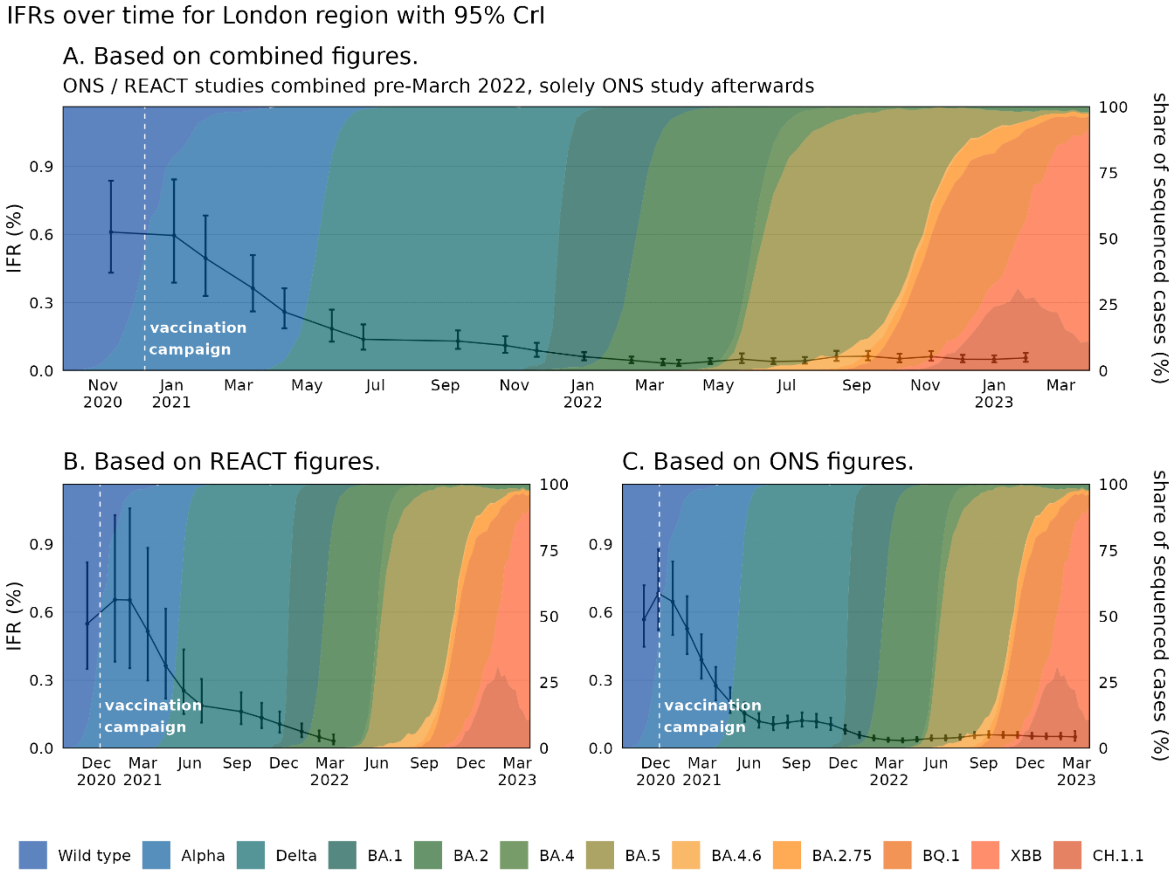


*Supplementary Figure 40 The Infection Fatality Risk for London. A: The posterior estimates of the median infection fatality risk for London, based on the combined REACT and ONS sampling, with 95% credible intervals. B: Posterior estimates of the median infection fatality risk for London, based on REACT sampling, with 95% credible intervals. C: Posterior estimates of the median infection fatality risk for London, based on ONS sampling, with 95% credible intervals. Not all estimates derived from the ONS CIS study have been plotted.*

## Supplementary Figure 41

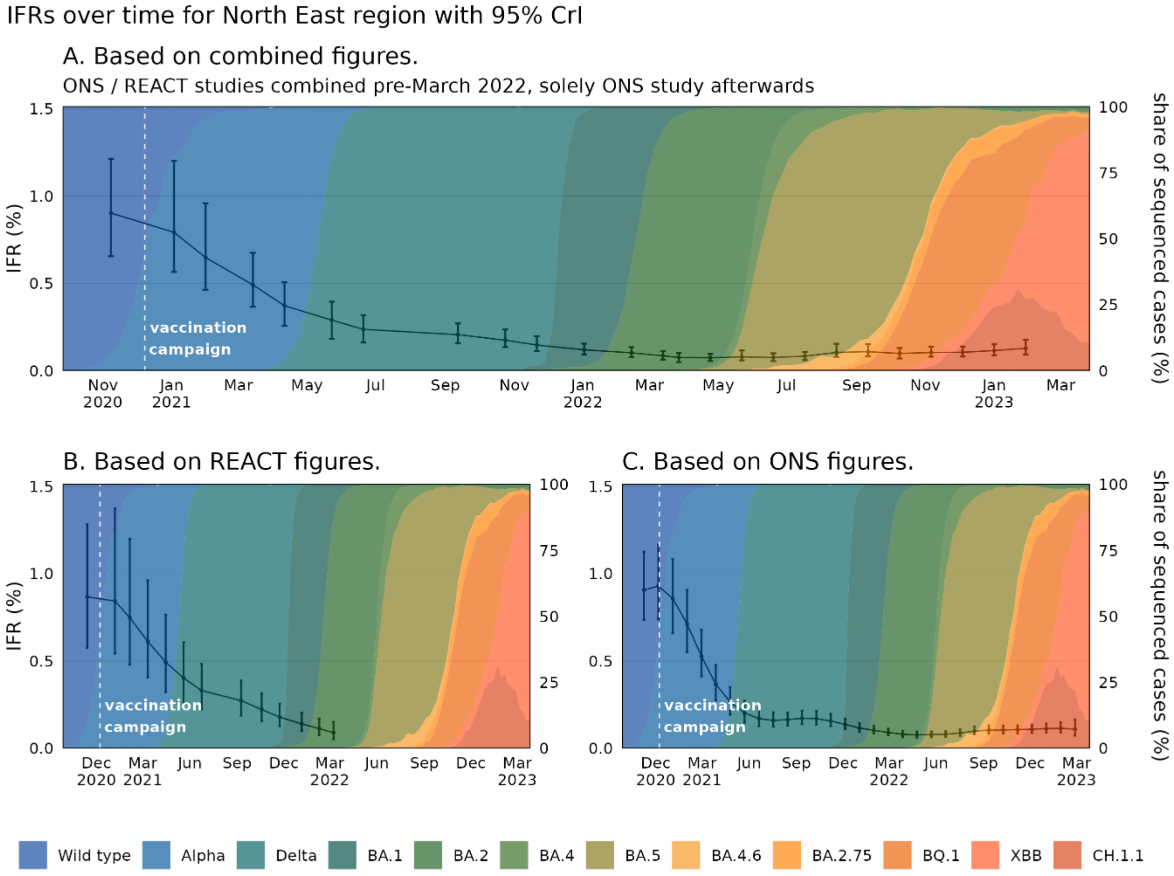


*Supplementary Figure 41 The Infection Fatality Risk for the North East. A: The posterior estimates of the median infection fatality risk for the North East, based on the combined REACT and ONS sampling, with 95% credible intervals. B: Posterior estimates of the median infection fatality risk for the North East, based on REACT sampling, with 95% credible intervals. C: Posterior estimates of the median infection fatality risk for the North East, based on ONS sampling, with 95% credible intervals. Not all estimates derived from the ONS CIS study have been plotted.*

## Supplementary Figure 42

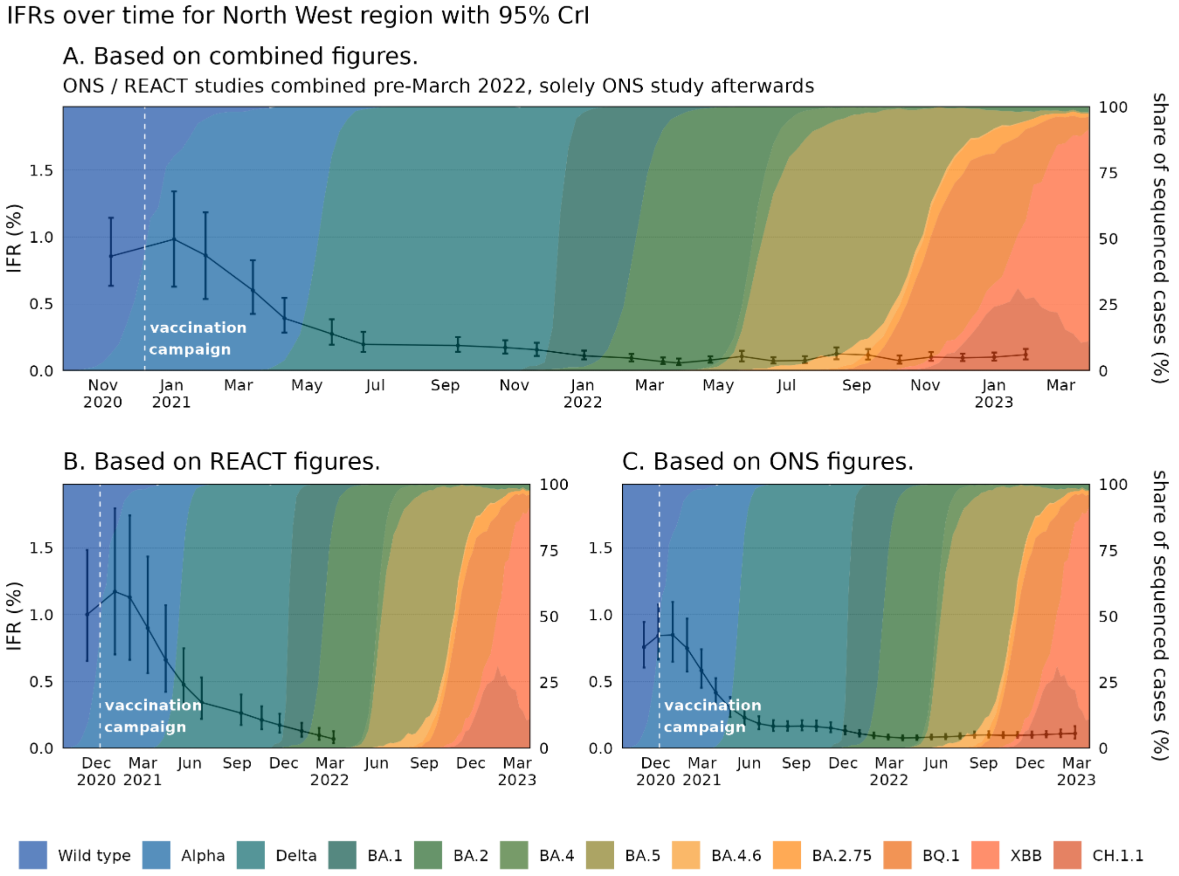


*Supplementary Figure 42 The Infection Fatality Risk for the North West. A: The posterior estimates of the median infection fatality risk for the North West, based on the combined REACT and ONS sampling, with 95% credible intervals. B: Posterior estimates of the median infection fatality risk for the North West, based on REACT sampling, with 95% credible intervals. C: Posterior estimates of the median infection fatality risk for the North West, based on ONS sampling, with 95% credible intervals. Not all estimates derived from the ONS CIS study have been plotted.*

## Supplementary Figure 43

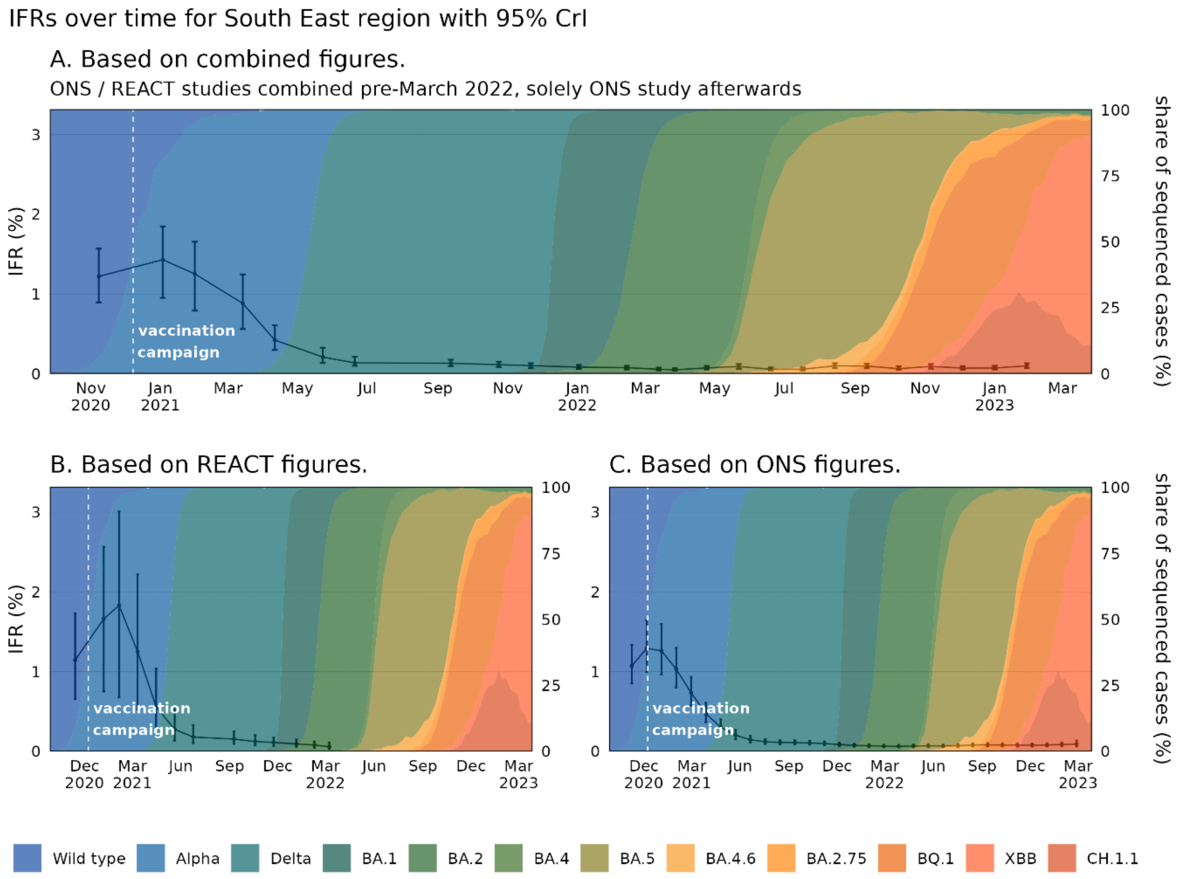


*Supplementary Figure 43 The Infection Fatality Risk for the South East. A: The posterior estimates of the median infection fatality risk for the South East, based on the combined REACT and ONS sampling, with 95% credible intervals. B: Posterior estimates of the median infection fatality risk for the South East, based on REACT sampling, with 95% credible intervals. C: Posterior estimates of the median infection fatality risk for the South East, based on ONS sampling, with 95% credible intervals. Not all estimates derived from the ONS CIS study have been plotted.*

## Supplementary Figure 44

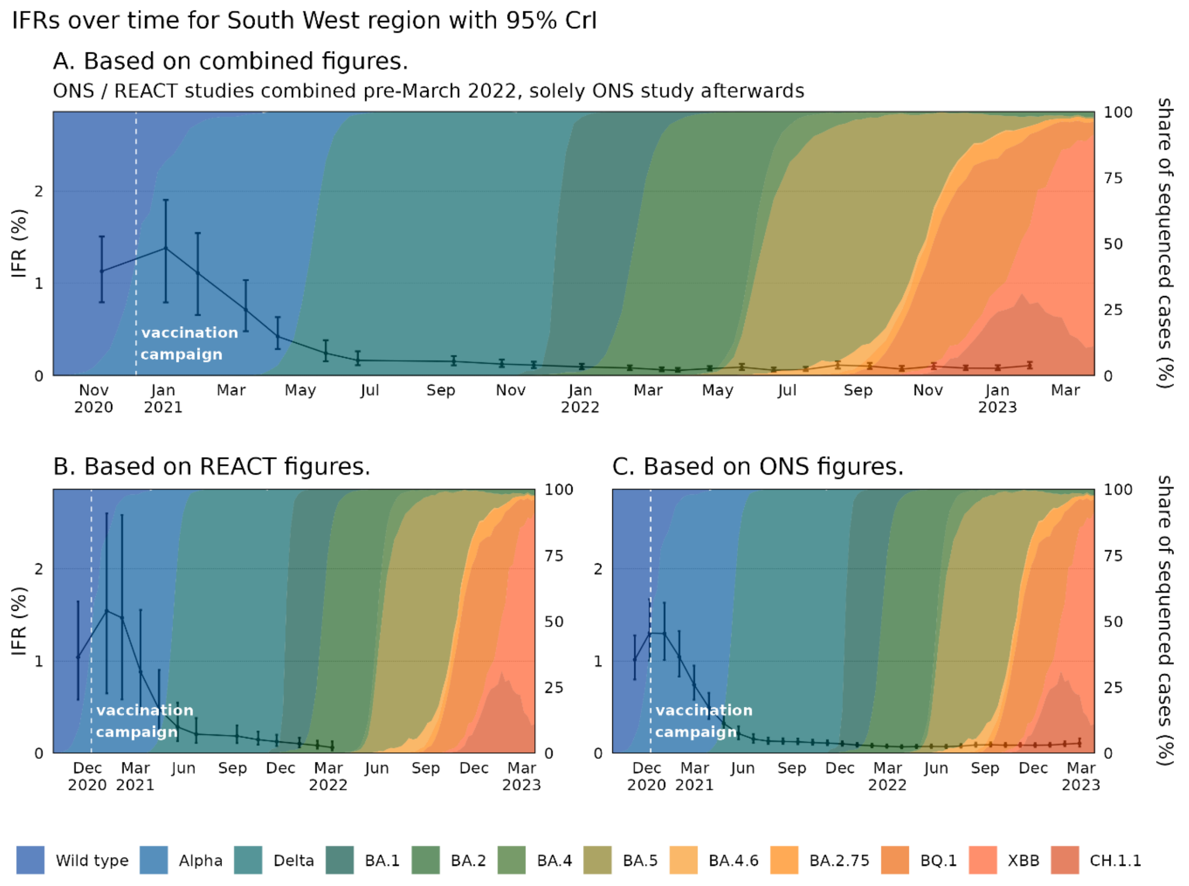


*Supplementary Figure 44 The Infection Fatality Risk for the South West. A: The posterior estimates of the median infection fatality risk for the South West, based on the combined REACT and ONS sampling, with 95% credible intervals. B: Posterior estimates of the median infection fatality risk for the South West, based on REACT sampling, with 95% credible intervals. C: Posterior estimates of the median infection fatality risk for the South West, based on ONS sampling, with 95% credible intervals. Not all estimates derived from the ONS CIS study have been plotted.*

## Supplementary Figure 45

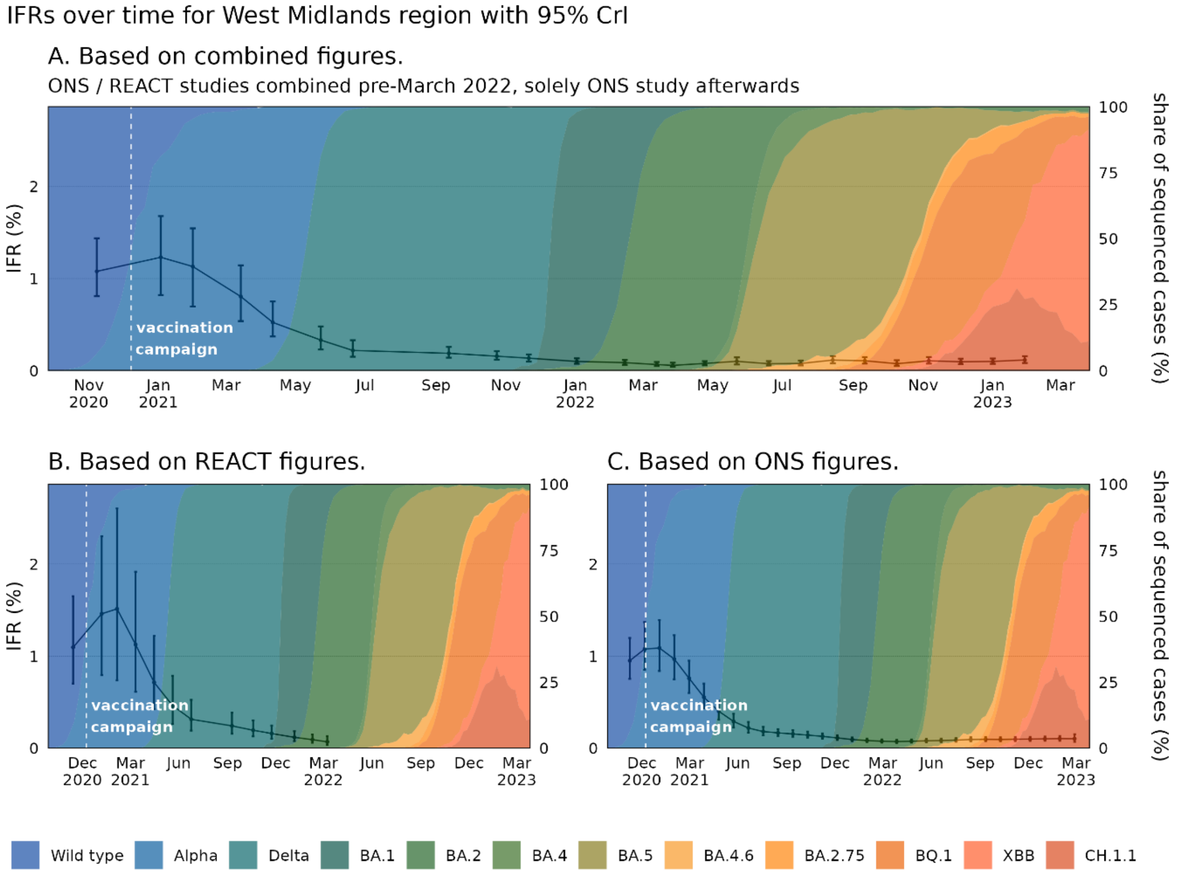


*Supplementary Figure 45 The Infection Fatality Risk for the West Midlands. A: The posterior estimates of the median infection fatality risk for the West Midlands, based on the combined REACT and ONS sampling, with 95% credible intervals. B: Posterior estimates of the median infection fatality risk for the West Midlands, based on REACT sampling, with 95% credible intervals. C: Posterior estimates of the median infection fatality risk for the West Midlands, based on ONS sampling, with 95% credible intervals. Not all estimates derived from the ONS CIS study have been plotted.*

## Supplementary Figure 46

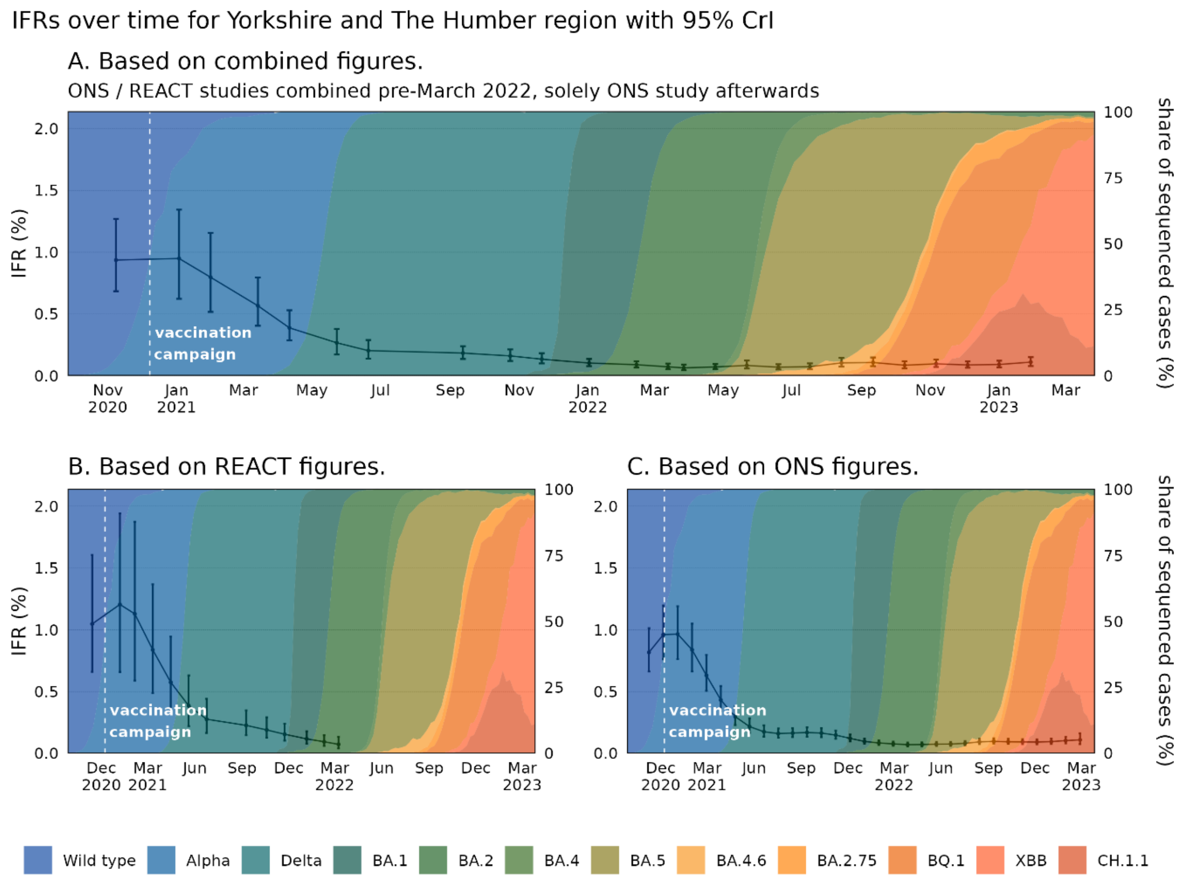


*Supplementary Figure 46 The Infection Fatality Risk for Yorkshire and the Humber. A: The posterior estimates of the median infection fatality risk for Yorkshire and the Humber, based on the combined REACT and ONS sampling, with 95% credible intervals. B: Posterior estimates of the median infection fatality risk for Yorkshire and the Humber, based on REACT sampling, with 95% credible intervals. C: Posterior estimates of the median infection fatality risk for Yorkshire and the Humber, based on ONS sampling, with 95% credible intervals. Not all estimates derived from the ONS CIS study have been plotted.*

**Supplementary Figure 47**

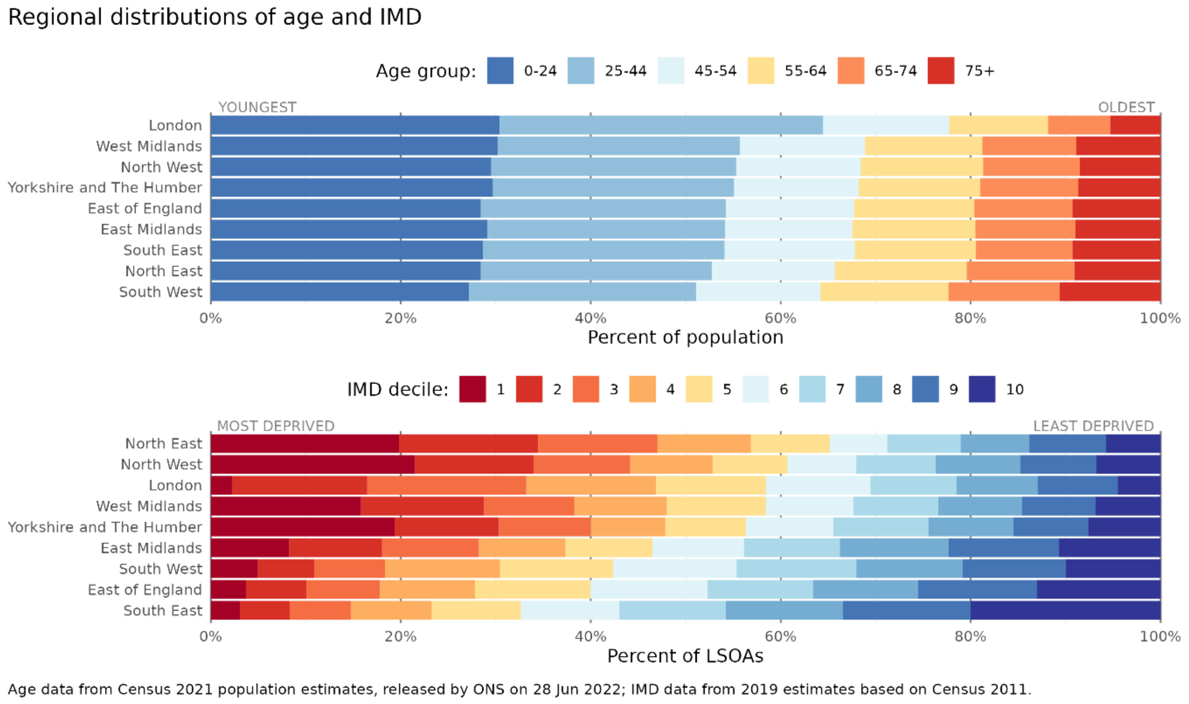


*Supplementary Figure 47 Top Panel: The age distribution for each English region, ranked by youngest to oldest (top to bottom). Bottom Panel: The index of multiple deprivation for each English region, ranked by most deprived to least deprived (top to bottom).*

## Supplementary Figure 48

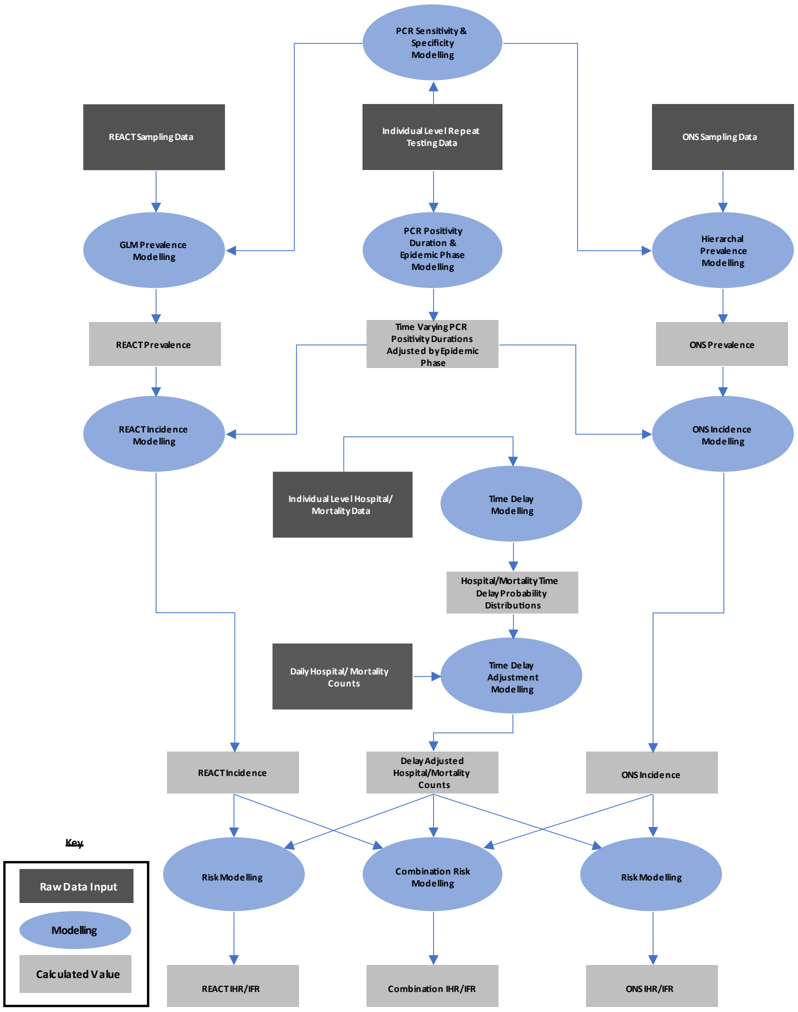


*Supplementary Figure 48: A process map of the methodological approach to calculate the infection hospitalisation/fatality risk. The methodology employs the ONS Coronavirus Infection Survey and the REACT 1 Survey sampling to calculate prevalence and incidence of SARS-CoV-2 with time delay adjusted hospitalisation/mortality data to estimate infection severity risk.*

## Supplementary Figure 49

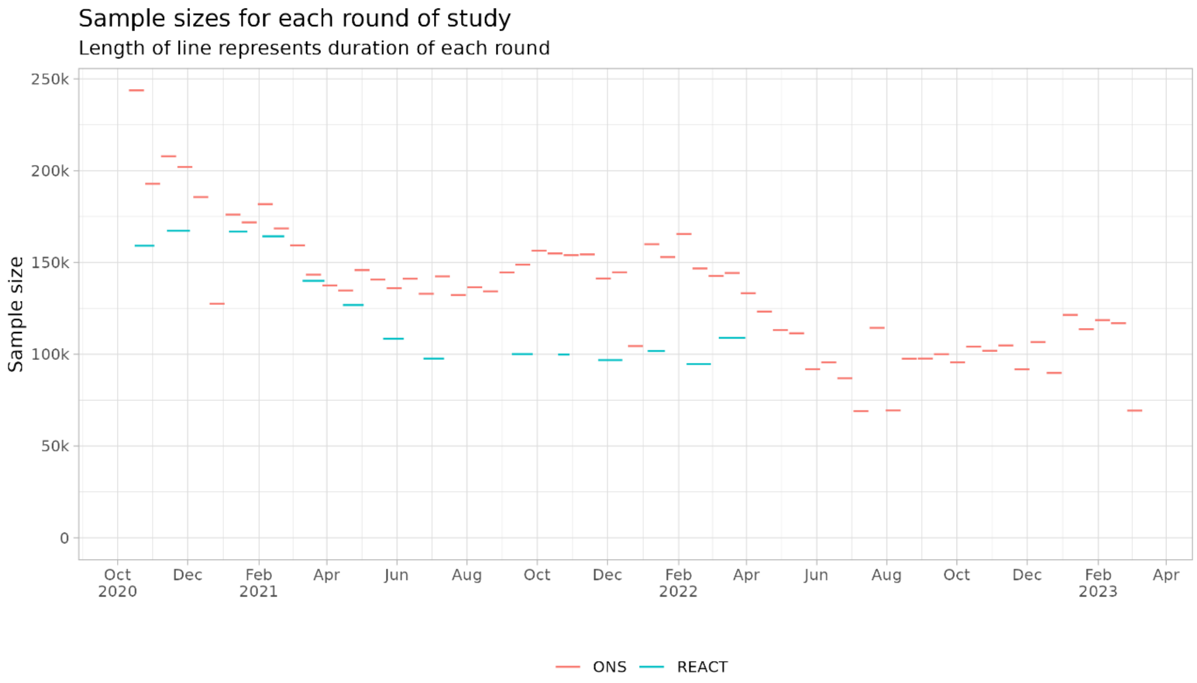


*Supplementary Figure 49: National sample sizes for each round of the ONS and REACT studies. The length of each line represents the length of each round. Note that ONS runs continuously, whilst REACT breaks between rounds.*

## Supplementary Figure 50

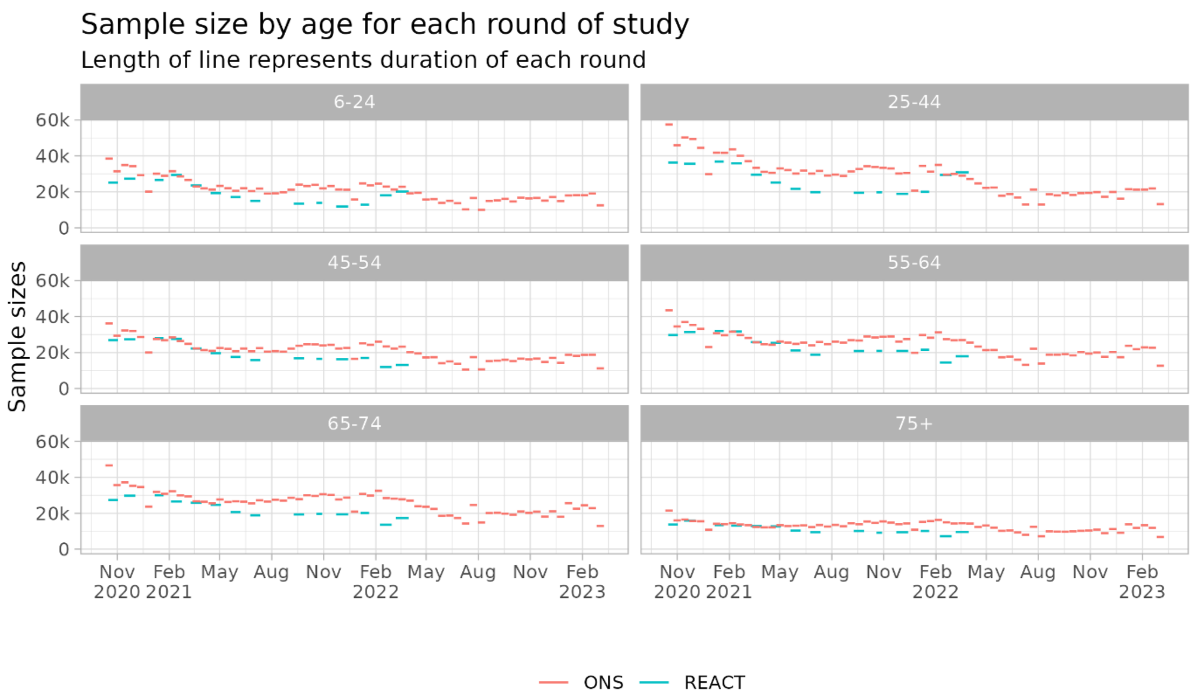


*Supplementary Figure 50: Sample sizes for each age group and round of the ONS and REACT studies. The length of each line represents the length of each round. Note that ONS runs continuously, whilst REACT had breaks between rounds.*

## Supplementary Figure 51

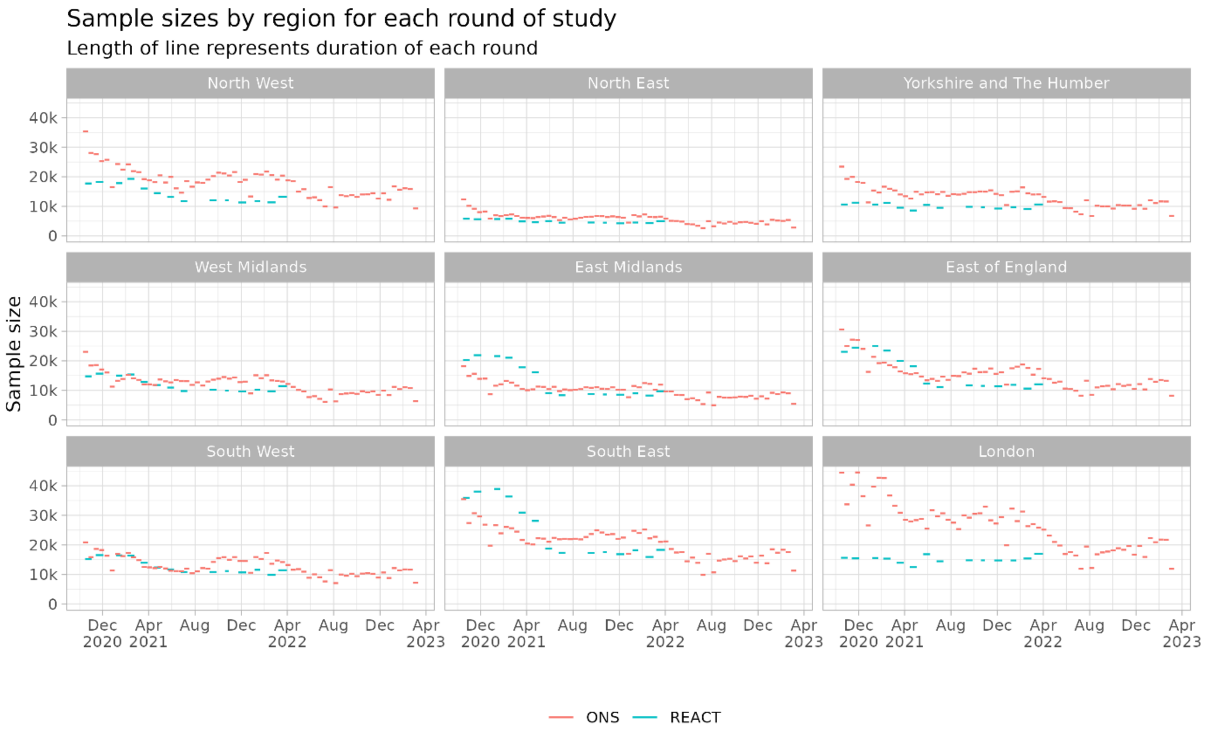


*Supplementary Figure 51: Sample sizes for each region and round of the ONS and REACT studies. The length of each line represents the length of each round. Note that ONS runs continuously, whilst REACT had breaks between rounds.*

**Supplementary Figure 52**

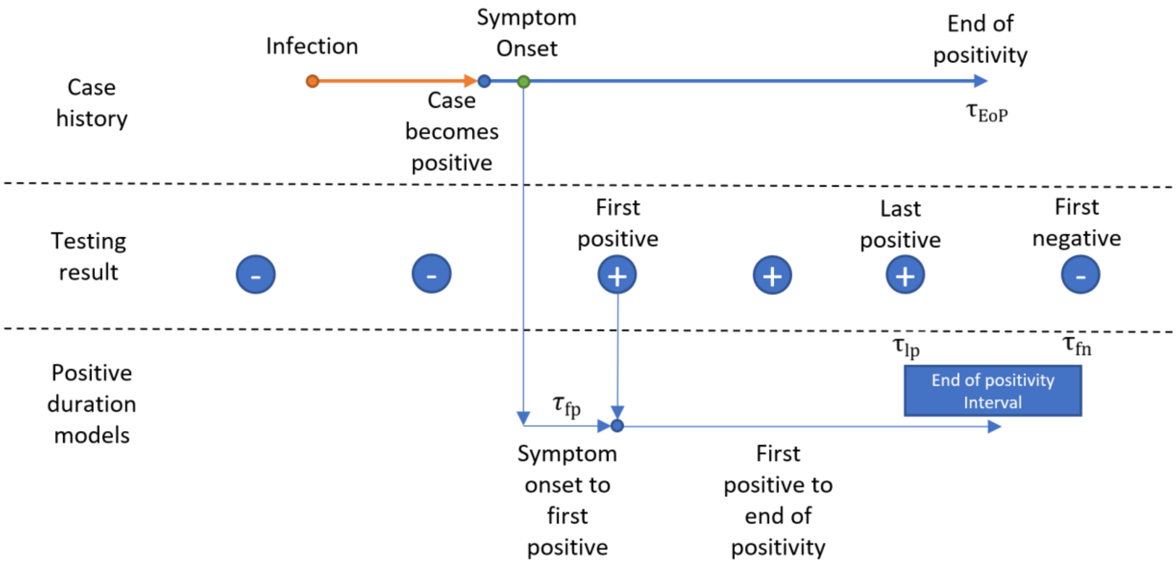


*Supplementary Figure 52: A schematic of the positivity duration calculation*
