# Supplementary material for: The real-time infection hospitalisation and fatality risk across the COVID-19 pandemic in England
Source: Nat Commun. 2024 May 31;15:4633. doi: 10.1038/s41467-024-47199-3 (PMC11143367; doi:10.1038/s41467-024-47199-3)
Supplement: Supplementary file 3 — Reporting Summary [file 41467_2024_47199_MOESM3_ESM.pdf]

nature portfolio

Corresponding author(s): Thomas Ward
Last updated by author(s): 01/03/2024

Reporting Summary

Nature Portfolio wishes to improve the reproducibility of the work that we publish. This form provides structure for consistency and transparency in reporting. For further information on Nature Portfolio policies, see our Editorial Policies and the Editorial Policy Checklist.

Statistics

For all statistical analyses, confirm that the following items are present in the figure legend, table legend, main text, or Methods section.

n/a	Confirmed	
☐	☒	The exact sample size (n) for each experimental group/condition, given as a discrete number and unit of measurement
☐	☒	A statement on whether measurements were taken from distinct samples or whether the same sample was measured repeatedly
☒	☐	The statistical test(s) used AND whether they are one- or two-sided<br>*Only common tests should be described solely by name; describe more complex techniques in the Methods section.*
☐	☒	A description of all covariates tested
☐	☒	A description of any assumptions or corrections, such as tests of normality and adjustment for multiple comparisons
☐	☒	A full description of the statistical parameters including central tendency (e.g. means) or other basic estimates (e.g. regression coefficient) AND variation (e.g. standard deviation) or associated estimates of uncertainty (e.g. confidence intervals)
☒	☐	For null hypothesis testing, the test statistic (e.g. F, t, r) with confidence intervals, effect sizes, degrees of freedom and P value noted<br>*Give P values as exact values whenever suitable.*
☐	☒	For Bayesian analysis, information on the choice of priors and Markov chain Monte Carlo settings
☒	☐	For hierarchical and complex designs, identification of the appropriate level for tests and full reporting of outcomes
☒	☐	Estimates of effect sizes (e.g. Cohen's d, Pearson's r), indicating how they were calculated

Our web collection on statistics for biologists contains articles on many of the points above.

Software and code

Policy information about availability of computer code

Data collection	AWS, R version 4.3.2
Data analysis	R version 4.3.2, cmdstanr 0.6.1

For manuscripts utilizing custom algorithms or software that are central to the research but not yet described in published literature, software must be made available to editors and reviewers. We strongly encourage code deposition in a community repository (e.g. GitHub). See the Nature Portfolio guidelines for submitting code & software for further information.

Data

Policy information about availability of data

All manuscripts must include a data availability statement. This statement should provide the following information, where applicable:

- Accession codes, unique identifiers, or web links for publicly available datasets
- A description of any restrictions on data availability
- For clinical datasets or third party data, please ensure that the statement adheres to our policy

The Office of National Statistics COVID Infection Survey (ONS CIS) data can be accessed through the Secure Research Service of the ONS. For all other datasets used in this study please contact the UKHSA.

UKHSA operates a robust governance process for applying to access protected data that considers:

• the benefits and risks of how the data will be used
• compliance with policy, regulatory and ethical obligations
• data minimisation
• how the confidentiality, integrity, and availability will be maintained
• retention, archival, and disposal requirements
• best practice for protecting data, including the application of 'privacy by design and by default', emerging privacy conserving technologies and contractual controls
Access to protected data is always strictly controlled using legally binding data sharing contracts.
UKHSA welcomes data applications from organisations looking to use protected data for public health purposes.
To request an application pack or discuss a request for UKHSA data you would like to submit, contact DataAccess@ukhsa.gov.uk.

Research involving human participants, their data, or biological material

Policy information about studies with human participants or human data. See also policy information about sex, gender (identity/presentation), and sexual orientation and race, ethnicity and racism.

Reporting on sex and gender	The study used all the participants from the Real-time Assessment of Community Transmission (REACT) and ONS CIS prevalence studies subset by age and region. The mortality and hospitalisations that were attributed to COVID-19 were also subset by region and age groups. This paper did not include a breakdown by sex and gender.
Reporting on race, ethnicity, or other socially relevant groupings	The study used all the participants from the REACT and ONS CIS prevalence studies subset by age and region. The mortality and hospitalisations that were attributed to COVID-19 were also subset by region and age groups. This paper did not include a breakdown by ethnicity or social characteristics.
Population characteristics	Mortality data, subset by age and geography, were sourced from the UKHSA COVID-19 death linelist. To prevent capturing deaths that were less likely to be linked to a COVID-19 infection we only included deaths that had occurred 60 days following a positive RT-PCR test.<br><br>Hospitalisations attributed to an infection with COVID-19 were collected from the NHSE&I situational report data, which exclude individuals that are not being treated for COVID-19. This includes:<br>a confirmed COVID-19 patient is any patient admitted to the trust who has recently (ie in the last 5 days) tested positive for COVID-19 following a polymerase chain reaction (PCR) test or LFD.<br>Patients who have been diagnosed via X-ray and assessment rather than a positive test should be counted as suspected (and not confirmed) COVID-19 patients.<br>Report a patient as a confirmed COVID-19 patient in the sitrep for as long as they are being treated as a COVID-19 patient – so either they are being treated for COVID-19 caused symptoms or the trust is still taking the precautions they would take with a COVID-19 positive patient.<br>A patient who has previously but not recently – (ie not in the last 5 days) had a positive COVID-19 test and is admitted for non-COVID-19 related treatment should not be counted as a confirmed COVID-19 patient.<br><br>Mortality data was available for the 9 English regions and hospital data is reported for the 7 NHS English Health regions.<br><br>The REACT survey included 2,512,551 tests conducted between May 2020 to March 2022. This survey was designed to capture a representative sample of the community in England.<br><br>The ONS CIS survey included 9,297,032 tests conducted between April 2020 to March 2023. This survey was designed to capture a representative sample of households in the United Kingdom.
Recruitment	This study uses data from the REACT and ONS CIS SARS-CoV-2 prevalence surveys. Both studies are described below<br><br>REACT was a repeat cross-sectional study that estimated SARS-CoV-2 prevalence in England from May 2020 until March 2022. The study aimed to sample between 95,000 to 175,000 individuals randomly for each survey round over the age of 5, which was updated from 100,000 to 150,000 individuals in the original study protocol. The study sent out recruitment letters on a 4–6-week basis to a randomised sample from the National Health Service patient register that aimed to be nationally representative. For children under the age of 18 the recruitment letters were sent to a parent or guardian. Individuals that chose to participate were sent throat and nasal swabs, which were sent for reverse transcription polymerase chain reaction (RT-PCR) testing. The participants were then invited to complete an online questionnaire, which included demographics, infection history, and behavioural topics. The REACT reports and study protocol have been published through Imperial College London and Welcome Open Research. Supplementary Figure 49-51 describes the sampling rate for each age group and region over time.<br><br>The ONS CIS study was produced in collaboration with the Wellcome Trust, University of Oxford, IQVIA, Lighthouse Laboratories, Joint Biosecurity Centre, UKHSA and the University of Manchester. The study began in April 2020 as a pilot and invited 20,000 households from the ongoing Labour Force Survey and those that had agreed previously to participate in the Opinions and Lifestyle Survey. Then in August 2020 the survey expanded to invite a randomised household sample from AddressBase [50]. The extended household study aimed to achieve around 150,000 swabs a fortnight in England between October 2020 to March 2023. The study requested that the entire household (over the age of 2) take a nose and throat swab, which was sent to the Lighthouse Laboratory for RT-PCR testing. Tests were conducted by home visits from a study worker and in April 2022 a proportion of participants of the study were asked to post their samples. At this time the number of swabs required reduced by 25% with an aim to swab 227,300 individuals every 28 days in England. From the 1st August 2022 the study collections had move to being entirely remote. The ONS reported an attrition rate of 0.62% in December 2020 that fluctuated between a high of 1.37% in July 2021 to the lowest rate of 0.32% in December 2021. The ONS study paused at the end of March 2023 with the intention to restart later in the year. Supplementary Figures 49-51 describes the sampling sizes

for each age group and region over time.

Ethics oversight

UKHSA have an exemption under regulation 3 of section 251 of the National Health Service Act (2006) to allow identifiable patient information to be processed to diagnose, control, prevent, or recognise trends in, communicable diseases and other risks to public health.

Note that full information on the approval of the study protocol must also be provided in the manuscript.

Field-specific reporting

Please select the one below that is the best fit for your research. If you are not sure, read the appropriate sections before making your selection.

☐ Life sciences ☒ Behavioural & social sciences ☐ Ecological, evolutionary & environmental sciences

For a reference copy of the document with all sections, see nature.com/documents/nr-reporting-summary-flat.pdf

Behavioural & social sciences study design

All studies must disclose on these points even when the disclosure is negative.

Study description

This paper calculates the real-time infection hospitalisation risk (IHR) and infection fatality risk (IFR) using sampling the Office for National Statistics Coronavirus Infection Survey (ONS CIS) and the Real-time Assessment of Community Transmission Survey between November 2020 to March 2023.

Research sample

Mortality data, subset by age and geography, were sourced from the UKHSA COVID-19 death linelist. To prevent capturing deaths that were less likely to be linked to a COVID-19 infection we only included deaths that had occurred 60 days following a positive RT-PCR test.

Hospitalisations attributed to an infection with COVID-19 were collected from the NHSE&I situational report data, which exclude individuals that are not being treated for COVID-19. This includes:
a confirmed COVID-19 patient is any patient admitted to the trust who has recently (ie in the last 5 days) tested positive for COVID-19 following a polymerase chain reaction (PCR) test or LFD.
Patients who have been diagnosed via X-ray and assessment rather than a positive test should be counted as suspected (and not confirmed) COVID-19 patients.
Report a patient as a confirmed COVID-19 patient in the sitrep for as long as they are being treated as a COVID-19 patient – so either they are being treated for COVID-19 caused symptoms or the trust is still taking the precautions they would take with a COVID-19 positive patient.
A patient who has previously but not recently – (ie not in the last 5 days) had a positive COVID-19 test and is admitted for non-COVID-19 related treatment should not be counted as a confirmed COVID-19 patient.

The REACT survey included 2,512,551 tests conducted between May 2020 to March 2022. This survey was designed to capture a representative sample of the community in England.

The ONS CIS survey included 9,297,032 tests conducted between April 2020 to March 2023. This survey was designed to capture a representative sample of households in the United Kingdom.

Sampling strategy

REACT was a repeat cross-sectional study that estimated SARS-CoV-2 prevalence in England from May 2020 until March 2022. The study aimed to sample between 95,000 to 175,000 individuals randomly for each survey round over the age of 5, which was updated from 100,000 to 150,000 individuals in the original study protocol. The study sent out recruitment letters on a 4–6-week basis to a randomised sample from the National Health Service patient register that aimed to be nationally representative. For children under the age of 18 the recruitment letters were sent to a parent or guardian. Individuals that chose to participate were sent throat and nasal swabs, which were sent for reverse transcription polymerase chain reaction (RT-PCR) testing. The participants were then invited to complete an online questionnaire, which included demographics, infection history, and behavioural topics. The REACT reports and study protocol have been published through Imperial College London and Welcome Open Research. Supplementary Figure 49-51 describes the sampling rate for each age group and region over time.

Please see the REACT Study Protocol (https://www.ncbi.nlm.nih.gov/pmc/articles/PMC8095190/) . As stated in the protocol, the REACT study was designed to be statistically powered to give information on every LTLA in England and this was under the assumption that prevalence in each Local Authority was independent. The study assumed that with 150,000 tests it would exclude prevalence of greater than 1.2% in each locality with a confidence of 95%, and this assumed a diagnostic sensitivity of 65% and a diagnostic specificity of 100%. The study estimated 100,000 tests would exclude prevalence above 1.7% per area for the same parameters.

The ONS CIS study was produced in collaboration with the Wellcome Trust, University of Oxford, IQVIA, Lighthouse Laboratories, Joint Biosecurity Centre, UKHSA and the University of Manchester. The study began in April 2020 as a pilot and invited 20,000 households from the ongoing Labour Force Survey and those that had agreed previously to participate in the Opinions and Lifestyle Survey. Then in August 2020 the survey expanded to invite a randomised household sample from AddressBase [50]. The extended household study aimed to achieve around 150,000 swabs a fortnight in England between October 2020 to March 2023. The study requested that the entire household (over the age of 2) take a nose and throat swab, which was sent to the Lighthouse Laboratory for RT-PCR testing. Tests were conducted by home visits from a study worker and in April 2022 a proportion of participants of the study were asked to post their samples. At this time the number of swabs required reduced by 25% with an aim to swab 227,300 individuals every 28 days in England. From the 1st August 2022 the study collections had move to being entirely remote. The ONS

reported an attrition rate of 0.62% in December 2020 that fluctuated between a high of 1.37% in July 2021 to the lowest rate of 0.32% in December 2021. The ONS study paused at the end of March 2023 with the intention to restart later in the year. Supplementary Figures 49-51 describes the sampling sizes for each age group and region over time.

Please see the ONS CIS Study Protocol (https://www.ndm.ox.ac.uk/covid-19/covid-19-infection-survey/protocol-and-information-sheets). As stated in the protocol:
"The target sample size for Phase I (around 10,000 households enrolled over one month, and around 21,000 individuals) was determined based on a conservative assumption that all members of the same household have the same infection status, and therefore each household should only be counted as one unit in the sample size calculation. If infection status varies within households, this will increase precision around our estimates. On balance, around 10,000 households in Phase I (around 21,000 individuals), and around 12,000 households (25,000 individuals) each subsequent month (3,000 per week) in Phase II, was considered to provide sufficient precision across England overall, particularly at lower prevalence rates which may be expected at earlier cross-sectional surveys, as well as the possibility of assessing evidence for variation within smaller but very important subgroups, including regions and Devolved Administrations (each targeting ~1,000 households per month, ~2,100 individuals per month). However, a major concern was the ability to monitor regions, rather than England as a whole, by October 2020 when the winter season of respiratory infections started, and monitoring for a possible "second wave" of infections was critical. Therefore, scaling up of recruitment from the end of July 2020 was designed to achieve similar numbers regionally as were originally available in England as a whole in Phase I, i.e. ~15,000-20,000 individuals with swab test results at least once each fortnight in each of the nine government office regions of England and also proportionate samples in Wales, Scotland, and Northern Ireland. Overall the swab target is therefore ~150,000 individuals with swab test results at least every fortnight from October in England."

Data collection	Data from the REACT survey was provided by the Imperial College London teams. Data from the ONS CIS survey was sourced from the Secure Research Service which is a part of the ONS. Mortality data was available to UKHSA from the mortality COVID-19 line-list and the hospitalisation data was sourced from NHS England.
Timing	The study period is the November 2020 to March 2023.
Data exclusions	Mortality data, subset by age and geography, were sourced from the UKHSA COVID-19 death line-list. To prevent capturing deaths that were less likely to be linked to a COVID-19 infection we only included deaths that had occurred 60 days following a positive RT-PCR test. Hospitalisation attributed to an infection with COVID-19 were collected from the NHSE&I situational report data, which exclude individuals that are not being treated for COVID-19.
Non-participation	The ONS CIS study (which was a longitudinal cohort study) reported an attrition rate of 0.62% in December 2020 that fluctuated between a high of 1.37% in July 2021 to the lowest rate of 0.32% in December 2021. The REACT study reported an overall response rate of 23·4%.
Randomization	The REACT and ONS CIS surveys were randomised SARS-CoV-2 prevalence surveys.<br><br>REACT was a repeat cross-sectional study that estimated SARS-CoV-2 prevalence in England from May 2020 until March 2022. The study aimed to sample between 95,000 to 175,000 individuals randomly for each survey round over the age of 5, which was updated from 100,000 to 150,000 individuals in the original study protocol. The study sent out recruitment letters on a 4–6-week basis to a randomised sample from the National Health Service patient register that aimed to be nationally representative. For children under the age of 18 the recruitment letters were sent to a parent or guardian. Individuals that chose to participate were sent throat and nasal swabs, which were sent for reverse transcription polymerase chain reaction (RT-PCR) testing. The participants were then invited to complete an online questionnaire, which included demographics, infection history, and behavioural topics. The REACT reports and study protocol have been published through Imperial College London and Welcome Open Research. Supplementary Figure 49-51 describes the sampling rate for each age group and region over time.<br><br>The ONS CIS study was produced in collaboration with the Wellcome Trust, University of Oxford, IQVIA, Lighthouse Laboratories, Joint Biosecurity Centre, UKHSA and the University of Manchester. The study began in April 2020 as a pilot and invited 20,000 households from the ongoing Labour Force Survey and those that had agreed previously to participate in the Opinions and Lifestyle Survey. Then in August 2020 the survey expanded to invite a randomised household sample from AddressBase [50]. The extended household study aimed to achieve around 150,000 swabs a fortnight in England between October 2020 to March 2023. The study requested that the entire household (over the age of 2) take a nose and throat swab, which was sent to the Lighthouse Laboratory for RT-PCR testing. Tests were conducted by home visits from a study worker and in April 2022 a proportion of participants of the study were asked to post their samples. At this time the number of swabs required reduced by 25% with an aim to swab 227,300 individuals every 28 days in England. From the 1st August 2022 the study collections had move to being entirely remote. The ONS reported an attrition rate of 0.62% in December 2020 that fluctuated between a high of 1.37% in July 2021 to the lowest rate of 0.32% in December 2021. The ONS study paused at the end of March 2023 with the intention to restart later in the year. Supplementary Figures 49-51 describes the sampling sizes for each age group and region over time.

Reporting for specific materials, systems and methods

We require information from authors about some types of materials, experimental systems and methods used in many studies. Here, indicate whether each material, system or method listed is relevant to your study. If you are not sure if a list item applies to your research, read the appropriate section before selecting a response.

Materials & experimental systems

n/a	Involved in the study
☒	☐ Antibodies
☒	☐ Eukaryotic cell lines
☒	☐ Palaeontology and archaeology
☒	☐ Animals and other organisms
☒	☐ Clinical data
☒	☐ Dual use research of concern
☒	☐ Plants

Methods

n/a	Involved in the study
☒	☐ ChIP-seq
☒	☐ Flow cytometry
☒	☐ MRI-based neuroimaging

Plants

Seed stocks	*Report on the source of all seed stocks or other plant material used. If applicable, state the seed stock centre and catalogue number. If plant specimens were collected from the field, describe the collection location, date and sampling procedures.*
Novel plant genotypes	*Describe the methods by which all novel plant genotypes were produced. This includes those generated by transgenic approaches, gene editing, chemical/radiation-based mutagenesis and hybridization. For transgenic lines, describe the transformation method, the number of independent lines analyzed and the generation upon which experiments were performed. For gene-edited lines, describe the editor used, the endogenous sequence targeted for editing, the targeting guide RNA sequence (if applicable) and how the editor was applied.*
Authentication	*Describe any authentication procedures for each seed stock used or novel genotype generated. Describe any experiments used to assess the effect of a mutation and, where applicable, how potential secondary effects (e.g. second site T-DNA insertions, mosiacism, off-target gene editing) were examined.*
